# The prognostic value of tumor mutational burden related 6-gene-based Risk Score in laryngeal cancer patients

**DOI:** 10.1186/s12903-022-02534-2

**Published:** 2022-11-17

**Authors:** Dong Yang, Juan Liu, Naibin Liu, Chunlei Yin, Huan Zhang, Jianhua Xu

**Affiliations:** 1 Department of Otolaryngology, Qingdao Chengyang People’s Hospital, Qingdao, 266109 Shandong China; 2 Department of Anesthesiology, Qingdao Chengyang People’s Hospital, Qingdao, 266109 Shandong China

**Keywords:** Laryngeal cancer (LC), Tumor mutation burden (TMB), Risk Score, Nomogram model

## Abstract

**Background:**

Laryngeal cancer (LC) is the second frequent malignant head and neck cancer around world, while LC patients’ prognosis is unsatisfactory. This study aims to investigate the prognostic value of tumor mutation burden (TMB)-related genes in LC.

**Methods:**

LC data was downloaded from The Cancer Genome Atlas and Gene Expression Omnibus databases. TMB values of all samples were calculated basing on mutation data. The differentially expressed genes (DEGs) between LC samples with distinct TMB were subjected to univariate and LASSO Cox regression analysis to build Risk Score. Immune cell infiltration analysis was conducted in CIBERSORT.

**Results:**

Between high and low TMB LC samples, we identified 210 DEGs. Of which, six optimal genes were included to construct Risk Score, comprising FOXJ1, EPO, FGF5, SPOCK1, KCNF1 and PSG5. High risk LC patients had significantly poorer overall survival than low risk patients. The nomogram model constructed basing on Risk Score and gender showed good performance in predicting LC patients’ survival probability.

**Conclusions:**

The prognostic Risk Score model, basing on six TMB-related genes (FOXJ1, EPO, FGF5, SPOCK1, KCNF1 and PSG5), was a reliable prognostic model to separate LC patients with different prognoses.

**Supplementary Information:**

The online version contains supplementary material available at 10.1186/s12903-022-02534-2.

## Background

Laryngeal cancer (LC) is the second most frequent head and neck cancer in the world, roughly accounting for one-third of all head and neck cancers [1]. It has been estimated that there will be 177,000 new cases and 95,000 LC related deaths worldwide in 2018 [2]. Moreover, the incidence of LC has remarkably increased in China recently and is higher in males than in females (22,500 new cases in males and 2,800 new cases in females in 2015) [3]. LC mainly originates from the epithelial tissue of the laryngeal mucosa and the majority of LC is well differentiated squamous cell carcinoma [4]. The main clinical manifestations of LC include hoarseness, foreign body sensation of the throat, and discomfort when swallowing, sometimes with irritating cough, blood in sputum and neck bump [5]. Many factors may lead to LC, including tobacco use, excessive alcohol consumption, virus infection and exposure to hazardous substances. Currently, surgery and/ or conservative treatment options (e.g., chemoradiotherapy and target therapy) are usually applied for LC patients based on the individual condition [6, 7]. The prognosis of LC patients diagnosed at early stage has been improved in accordance to the development of treatment technology. However, owing to the high rates of recurrence and tendency of developing resistance to clinical therapy, the prognosis of advanced LC patients remains poor, with approximately 60% 5-year overall survival (OS) rate [8, 9]. Although many clinical variables have been considered as major prognostic factors, comprising nodal involvement, the site and volume of the primary tumor, and stages of tumor, their invasive procedures inevitably exert negative impacts on the patients [10]. As the development of medical laboratory techniques, several studies have identified some less invasive prognostic indicators, such as immune-related genes, inflammatory-related genes, and glycolysis-related genes [11], also indicating prognostic values for LC patients. These indicators are proven to be reliable prognostic biomarkers, but more indicators are still needed to explore more accurate prediction for the prognosis of cancer patients.

Mutations are of benefit to evolution as a source of genetic diversity, while higher than normal rates of mutations (genomic instability) may have serious consequences for some diseases, especially various cancers. Tumor mutational burden (TMB), as a quantitative biomarker, is usually defined as the number of mutations per coding area of genomic sequence, reflecting the tumor mutation quantity. These mutations are processed into neoantigens, then they are presented to T cells, so higher TMB level may incline to harbor more neoantigens as targets for activated immune cells, which thereby increases the chances for T cell recognition and enhances anti-tumor effects [12]. On the other hand, tumors could inhibit the reactivity of T cells via immune checkpoints, to evade immune eradication. A variety of studies have shown that compared with the patients with lower TMB levels, patients with higher TMB levels have greater response rates after immune checkpoint inhibitor therapy and experience longer survival time [13]. Results of prior studies have suggested that high TMB level is emerging as a novel predictive biomarker of sensitivity to immunotherapy in diverse cancers [14], while the prognostic values of TMB-related genes for LC patients have not been thoroughly explored yet. The whole exome sequencing (WES) is a golden standard for estimation of TMB in clinic, but the panel sequencing-based estimates of TMB has largely replaced WES-derived TMB due to the high prices.

In this study, we aimed to identify TMB-related genes in LC and to explore the connection between these TMB-related genes and the prognosis of LC patients. We hope to find novel predictive biomarkers to assist screening of LC patients with different prognosis.

## Material and methods

### Data sources

We downloaded 120 mRNA expression profiles with corresponding complete clinical information and 82 whole exome somatic mutation profiles of LC patients from the Cancer Genome Atlas (TCGA, https://tcga-data.nci.nih.gov/tcga/) database to construct LASSO Cox model, and the clinical information was shown on Table 1. We also downloaded GSE27020 dataset, comprised of expression profiles of 109 LC patients with complete clinical information, from the Gene Expression Omnibus (GEO, https://www.ncbi.nlm.nih.gov/geo/) database to verify the prognostic model. The patients’ expression profiles were obtained by Affymetrix Human Genome U133A Array.

**Table 1:** Clinicopathological characteristics of LC patients from TCGA database

**Characteristics**		Patients (*N* = 120)	
		No.	%
Gender	Female	23	19.17%
	Male	97	80.83%
Age	≤ 62(Median)	65	54.17%
	> 62(Median)	55	45.83%
Stage	I	3	2.50%
	II	11	9.17%
	III	27	22.50%
	IV	75	62.50%
	Not reported	4	3.33%
Survival Time	Long(> 5 years)	18	15.00%
	Short(< 5 years)	102	85.00%
OS status	Dead	53	44.17%
	Alive	67	55.83%
Alcohol	NO	42	35.00%
	YES	76	63.33%
	Not reported	2	1.67%
Tobacco	NO	7	5.83%
	YES	110	91.67%
	Not reported	3	2.50%

### TMB value calculation

TMB referred to the total number of mutated genes (somatic genes with coding errors, base substitutions, or deletion errors) per coding area of genomic sequence (Mb). The TMB values of LC samples were calculated basing on the mutation data (files), using  package of R.

### Differential expression analysis

Differential expression analysis was performed based on the limma package of R programming software (version 4.1.0, the same below), with the thresholds of |Log_2_FC|> 2 and multiple testing adjusted *p* value ≤ 0.05 to screen the significantly differential expression genes (DEGs). And *p* value was adjusted by the Benjamini and Hochberg (BH) method.

### Functional enrichment analysis

Built-in functions (enrichGO and enrichKEGG) in “ClusterProfiler” function package of R programming software were used to perform Gene ontology (GO) terms and Kyoto Encyclopedia of Genes and Genomes (KEGG) pathways enrichment analyses on the DEGs. To screen the significantly enriched GO terms and KEGG pathway, the BH method adjusted *p* value < 0.05 was used as the threshold. The GO enrichment was conducted by the org.Hs.eg.db of Bioconductor (version 3.13) and KEGG enrichment was conducted by the Release 100.0 of KEGG database.

### Calculation of risk score

Univariate Cox regression analysis was applied to the 120 LC patients based on the expression values of screened DEGs, with the *p* value < 0.01 as threshold to screen genes significantly associated with overall survival (OS) of LC. To further optimize the genes, LASSO Cox regression analysis was performed on the TCGA dataset using the glmnet package in R programming software. Risk Score of each patient was calculated by the following formula using the screened TMB-related genes:

$${\text{Risk Score}} = \sum_{{{\text{i}} = {1}}}^{{\text{n}}} {\text{Coef}}_{{\text{i}}} {\text{*X}}_{{\text{i}}} {,}$$

was the LASSO Cox coefficient of gene i and Xi was the relative expression of gene i (mRNA expression in this study). Survival, and two-sided log-rank test of R package were used to determine the values of Risk Score, and the patients in the GEO dataset were classified into low-risk and high-risk groups according to the median of the Risk Score.

### Survival analysis

Kaplan–Meier survival analysis was used to assess the OS rates of LC patients in GEO dataset. We used the survival and packages of R programming software and the significance of OS rate difference between different groups was examined by the log-rank test. Multivariate Cox regression model was used to test the independence of Risk Score in predicting the prognosis of LC from other clinical factors.

### Construction of nomogram model

Nomograms are widely used for prognosis of many cancers, mainly because they can simplify statistical predictive models. We used the package (http://CRAN.R-project.org/package=rms) of R programming software to construct a nomogram using the independent prognostic factors identified by the multivariate Cox analysis to predict the OS rates of patients in 1 year, 3 years and 5 years. A calibration curve of nomogram was drawn to observe the correlation between the living probability of LC patients and actual survival probability.

### Immune cell infiltration analysis

Finally, the immune cell infiltration analysis was performed in LC samples with distinct Risk Score. The relative proportions of various immune cells (totally 22 types) in each LC sample were calculated using software CIBERSORT [15]. All estimated proportions of immune cells in each sample summed up to 1.

## Results

### The DEGs between high TMB and low TMB LC samples

**Fig. 1:**
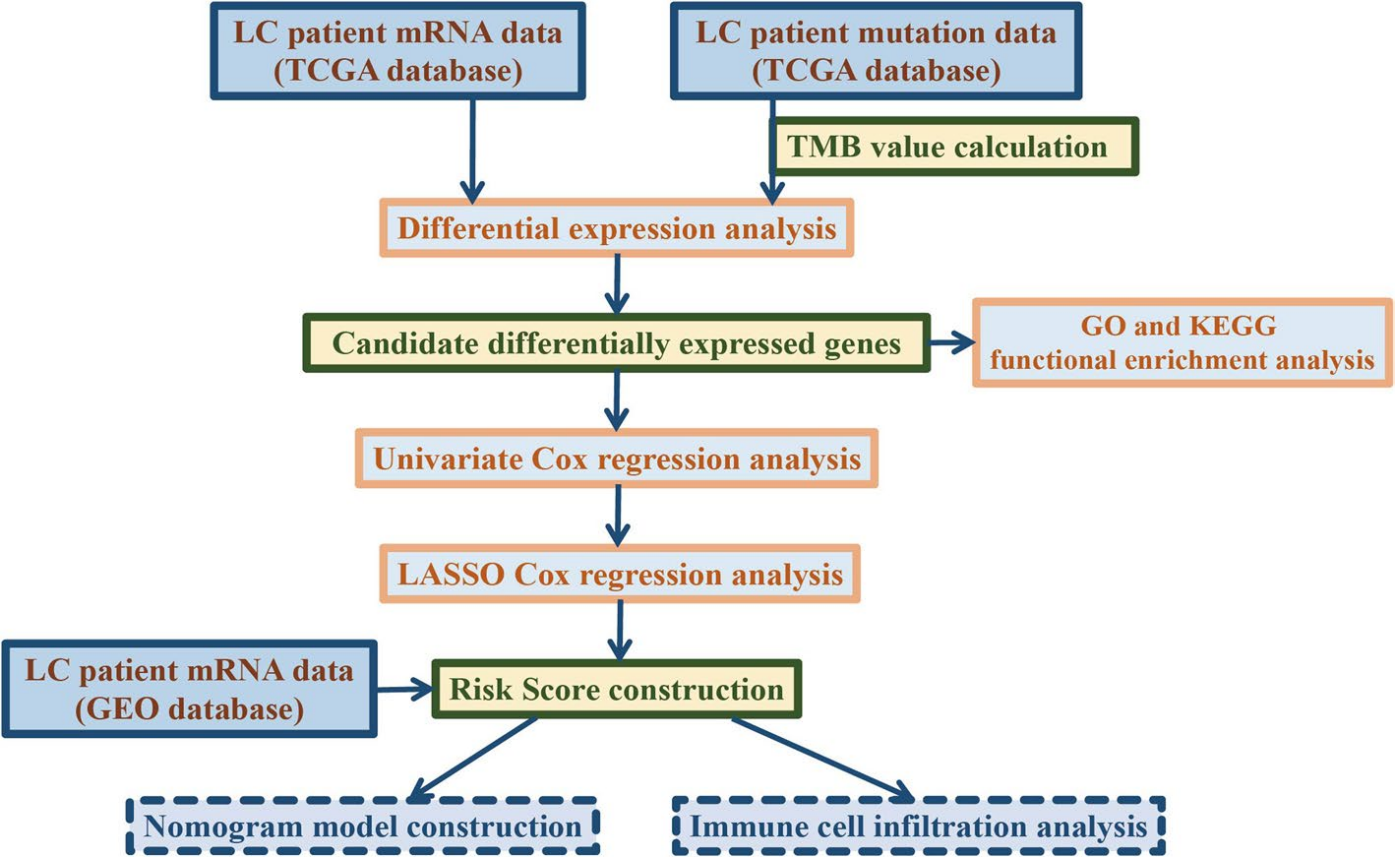
The flowchart of this work

**Fig. 2:**
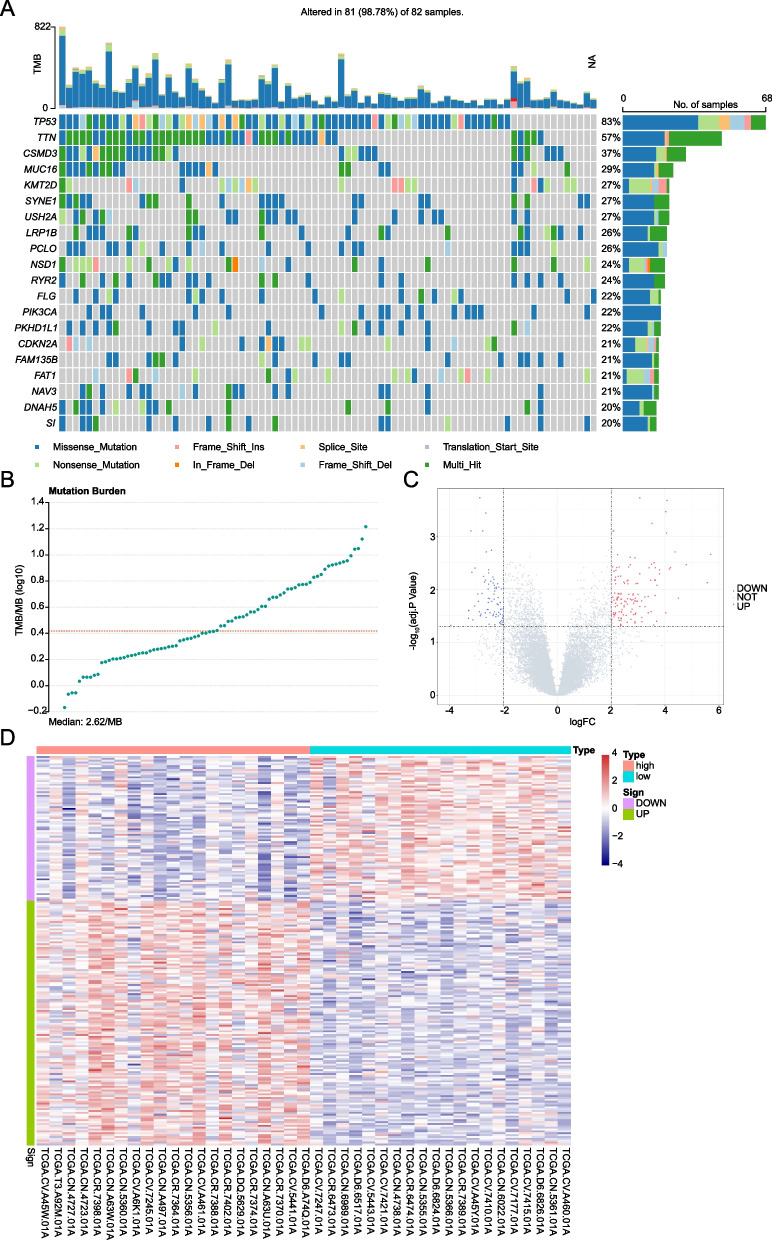
Mutated profiling in TCGA and differential expression analysis. **A** Landscape of mutation profiles of top 20 genes. The waterfall plot shows the mutation information of each gene. **B** Distribution graph of TMB. The horizontal axis is TMB values and the vertical axis is TMB values of log base 10. **C** Volcano plot of DEGs. The horizontal axis is Log_2_FC and the vertical axis is log_10_ (adj. *p*. value). The blue dots are downregulation genes and the red dots are upregulation genes. **D** Heatmap of DEGs. The horizontal and vertical axes are samples and different genes respectively. The red represents high expression of genes and the blue represents low expression of genes. The green is upregulation genes and the purple is downregulation genes

To explore the prognostic value TMB-related genes in LC patients, the flowchart of our present study was displayed in Fig. 1. In the 82 LC samples with mutation data (from TCGA database), TP53, TTN and CSMD3 were the top 3 most frequently mutated genes (Fig. 2A). The TMB is the significant characteristic of genomic instability, and TMB level was used to define the genomic instability in this work. Then TMB values of these 82 LC patients were calculated basing on the mutation data. After sorting in ascending order, totally 42 patients with top 25% (≤ 1.765) or bottom 25% (≥ 5.495) TMB values were found, which were divided as low TMB (≤ 1.765) and high TMB (≥ 5.495) group (Fig. 2B). Eventually 41 LC patients with mRNA expression profiles (one patient without expression profile was removed) were screened for the subsequent analysis. Compared to the low TMB group, there were 210 DEGs in the high TMB group, including 132 upregulated genes and 78 downregulated genes (Fig. 2C). The expression levels of these DEGs significantly differed between the high TMB and low TMB groups (Fig. 2D).

### Significant functional GO terms and KEGG pathways

**Fig. 3:**
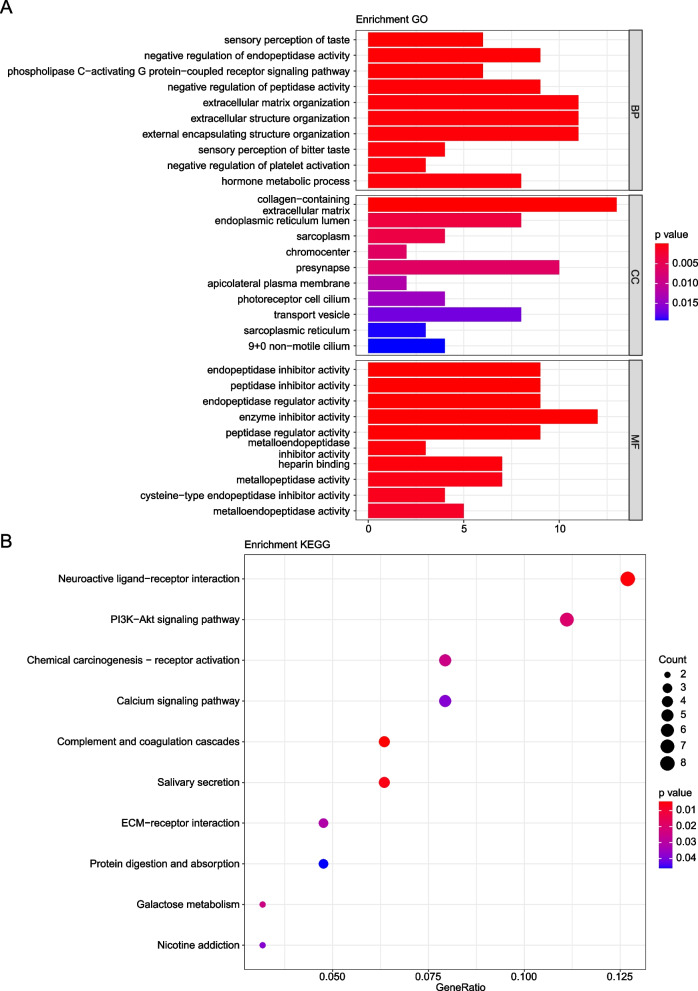
Results of functional enrichment analysis. **A** Top 10 enriched GO terms which are significantly enriched. The horizontal axis is the number of genes which are enriched in the term. The vertical axis is the name of the GO terms (BP: biological process; CC: cellular component; MF: molecular function). **B** Top 10 KEGG pathways. The horizontal axis is the number of genes which are enriched in the term. The vertical axis is the name of the KEGG pathways

To obtain more functional information about the DEGs between high and low TMB LC patients, the above 210 DEGs were then subjected to the functional enrichment analysis. Our results showed that these 210 genes were significantly enriched in 245 biological process (BP) terms (including sensory perception of taste), 21 cellular component (CC) terms (including collagen containing extracellular matrix), and 49 molecular function (MF) terms (including endopeptidase inhibitor activity), as well as 11 KEGG pathways including complement and coagulation cascades (The pathways were obtained basing on KEGG [16–18]). These 210 genes were significantly enriched in the pathways involving the tumor heterogeneity. The top 10 significantly enriched GO terms and KEGG pathways were shown in Fig. 3A-B, respectively. Meanwhile, the full lists of enriched GO terms and KEGG pathways were shown in Table S1 and Table S2, separately.

### Construction and validation of prognostic Risk Score model

**Fig. 4:**
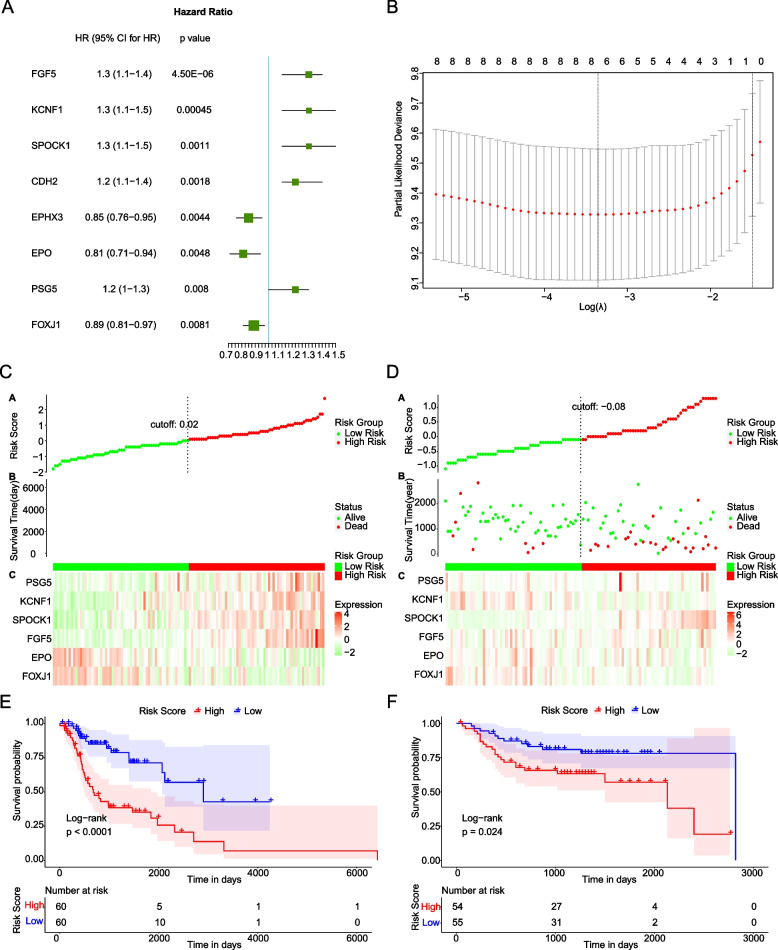
Construction of prognostic model for LC. **A** Forrest plot of univariate Cox regression analysis showing 8 genes identified as prognostic factors for LC. HR is short for Hazard ratio and 95%CI is 95% confidence interval. **B** The graph of the LASSO Cox regression analysis to determine the tuning parameter lambda. The horizontal axis is the log(lambda) and the vertical axis is partial likelihood Deviance the lowest value of which is the best Lambda. **C** Risk Score map. The vertical dotted line is the median of Risk Score. The red dots represent high-risk patients and the blue dots represent low-risk patients. **D** Risk Score map in GEO dataset. The vertical dotted line is the median of Risk Score. The red dots represent high-risk patients and the blue dots represent low-risk patients. **E** Kaplan–Meier survival curve in TCGA dataset. The horizontal and vertical axes are time and survival probability. Different colors represent different groups. *p* value is determined by the log-rank test. **F** Kaplan–Meier survival curve in GEO dataset. The horizontal and vertical axes are time and survival probability. Different colors represent different groups. *p* value is determined by the log-rank test

The mRNA expressions of the 210 TMB-related DEGs were used as continuous variable to perform univariate Cox regression analysis on the 120 LC patients from TCGA database. Hazard ratio (HR) of each gene was calculated. Then p value < 0.01 was used as threshold to screen prognosis related genes. A total of 8 genes were significantly correlated with the OS of LC patients, including FGF5, KCNF1, SPOCK1, CDH2, EPHX3, EPO, PSG5, and FOXJ1 (Fig. 4A).

Those eight TMB-related genes were further optimized to six genes (FOXJ1, EPO, FGF5, SPOCK1, KCNF1 and PSG5) based on the corresponding lambda value of different genes from LASSO Cox analysis (Fig. 4B, the smallest lambda value).

Then mRNA expressions were weighted with LASSO Cox regression coefficient to construct prognostic Risk Score model as follows: Risk Score = (-0.005280855) * Express Value of FOXJ1 + (-0.142224223) * Express Value of EPO + (0.176618430) * Express Value of FGF5 + (0.065174944) * Express Value of SPOCK1 + (0.047590189) * Express Value of KCNF1 + (0.017885014) * Express Value of PSG5. Patients in the TCGA and GSE27020 datasets were assigned to high- and low-risk groups according the median of Risk Scores. After integrating the correlation between the order of estimated Risk Score and survival time (Fig. 4C-D), we found that the survival time (measured by day and year) of the low-risk group was higher than that of the high-risk group and the death toll of high-risk group was higher than that of the low-risk group. The green and red dots represent alive and dead patients respectively. The OS of high-risk group were proved to be significantly lower than those of the low-risk group in the TCGA dataset (*p* < 0.0001) and GEO validation set (*p* = 0.024) by Kaplan–Meier analysis (Fig. 4E-F). The results indicated that Risk Score, calculated by the prognostic model constructed by the 6 TMB-related genes (FOXJ1, EPO, FGF5, SPOCK1, KCNF1 and PSG5), may effectively stratify LC patients with different prognosis.

### Risk Score represents an independent prognosis signature of LC Patients

**Fig. 5:**
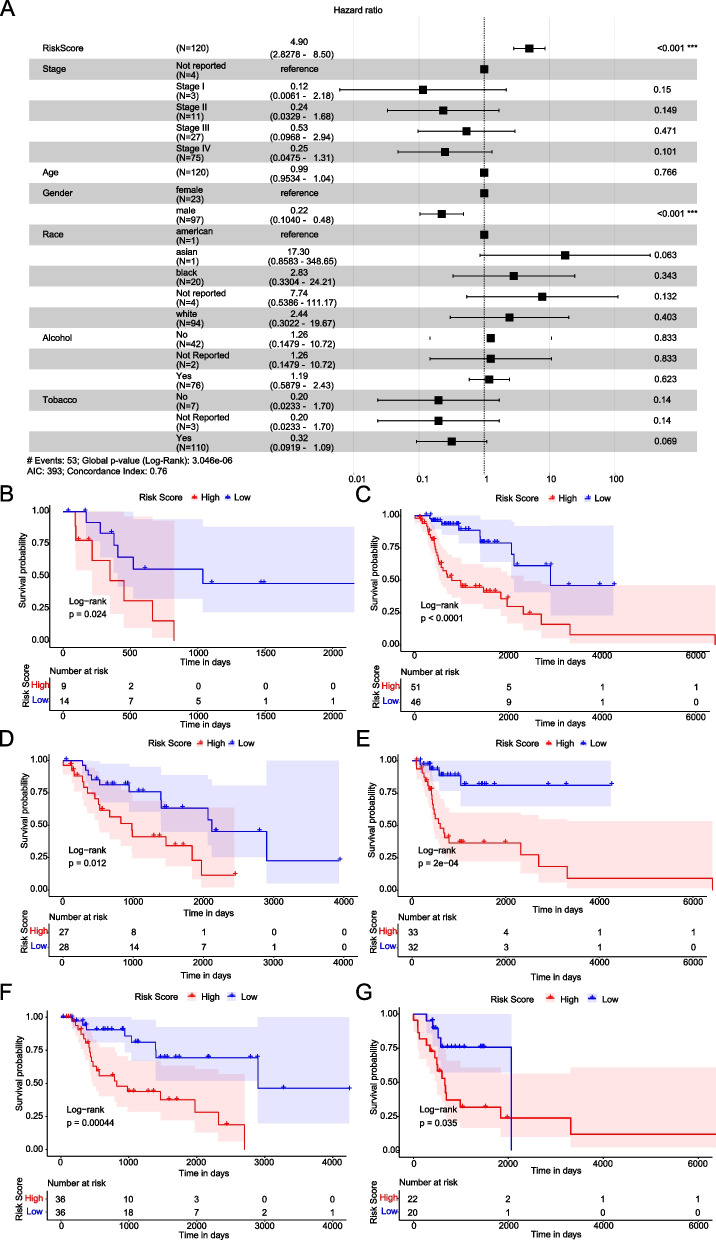
Risk Score is an independent prognostic biomarker for LC patients. **A** Forrest plot of multivariate Cox regression analysis. Compared to patients in the reference groups, patients with Hazard ratio > 1 have higher death risk and patients with Hazard ratio < 1 have lower death risk. **B**-**C** Kaplan–Meier survival curve of the female and male subgroups. **D**-**E** Kaplan–Meier survival curve of the ≤ 62 and > 62 years old subgroups. **F**-**G** Kaplan–Meier survival curve of with and without alcohol history

Seven factors, comprised of age, gender, TNM stage, race, alcohol history, tobacco history and Risk Score, were included to perform multivariate Cox regression analysis to test the independence in prognosis estimation of Risk Score. We found that Risk Score (*p* < 0.001) and gender (*p* < 0.001) were significantly correlated with OS, which indicated that they might be reliable prognostic factors (HR = 4.90, 95%, CI: 2.827—8.50, *p* < 0.001) (Fig. 5A).

To further investigate whether the prognostic value of Risk Score in LC patients was independent from other clinical factors, the stratification analysis was conducted based on the age, gender, and alcohol history. The results showed that the survival probability of the high-risk LC patients was significantly lower than that of the low-risk patients in both the female (*p* = 0.024) and male (*p* < 0.0001) subgroups (Fig. 5B-C). The same results was found in the younger (≤ 62 years old) (*p* = 0.012), older (> 62 years old) (*p* = 2 * 10^–6^) subgroups (Fig. 5D-E), and with and without alcohol history LC patients (Fig. 5F-G). These indicated that Risk Score could be used as an independent prognostic indicator for LC patients.

### Nomogram model to predict the prognosis of LC patients

**Fig. 6:**
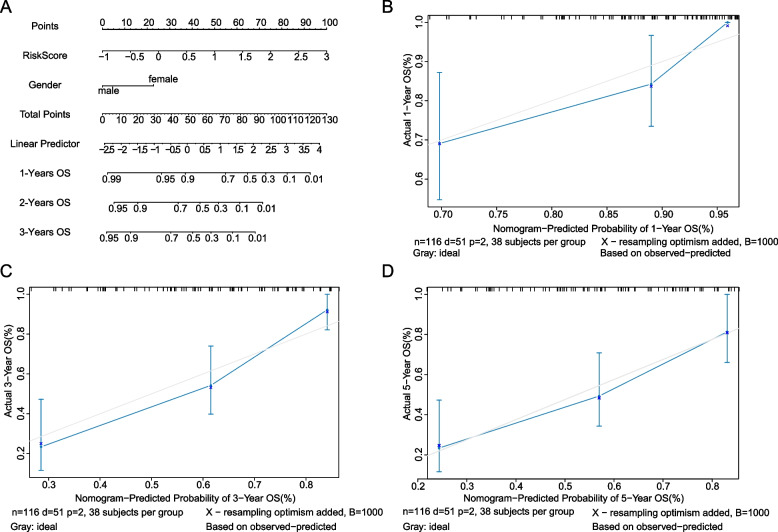
Nomogram to predict the survival status of LC patients. **A** Nomogram based on the Risk Score and gender for 1-, 3- and 5-year OS prediction. **B**-**D **Calibration curves of nomogram in 1-, 3- and 5-year. The x axis is the predicted probability of nomogram and the y axis is the actual survival probability

Two independent prognostic factors (Risk Score and gender) were used to construct the nomogram model (Fig. 6A). To get the points of Risk Score and gender, two lines were drawn in the nomogram and the sum of the two points was located on the “Total Points” axis. The 1-year, 3-year and 5-year OSs were obtained by drawing a line from the “Total Points” axis. The adjusted curves were close to the ideal curve (a 45 degree line with the slop 1 through the origin), which indicated that the nomogram constructed by Risk Score and gender was in good performance in predicting the OSs in 1 year, 3 years and 5 years (Fig. 6B-D).

### The infiltration ratios of immunosuppressive cells are higher in the high-risk group

**Fig. 7:**
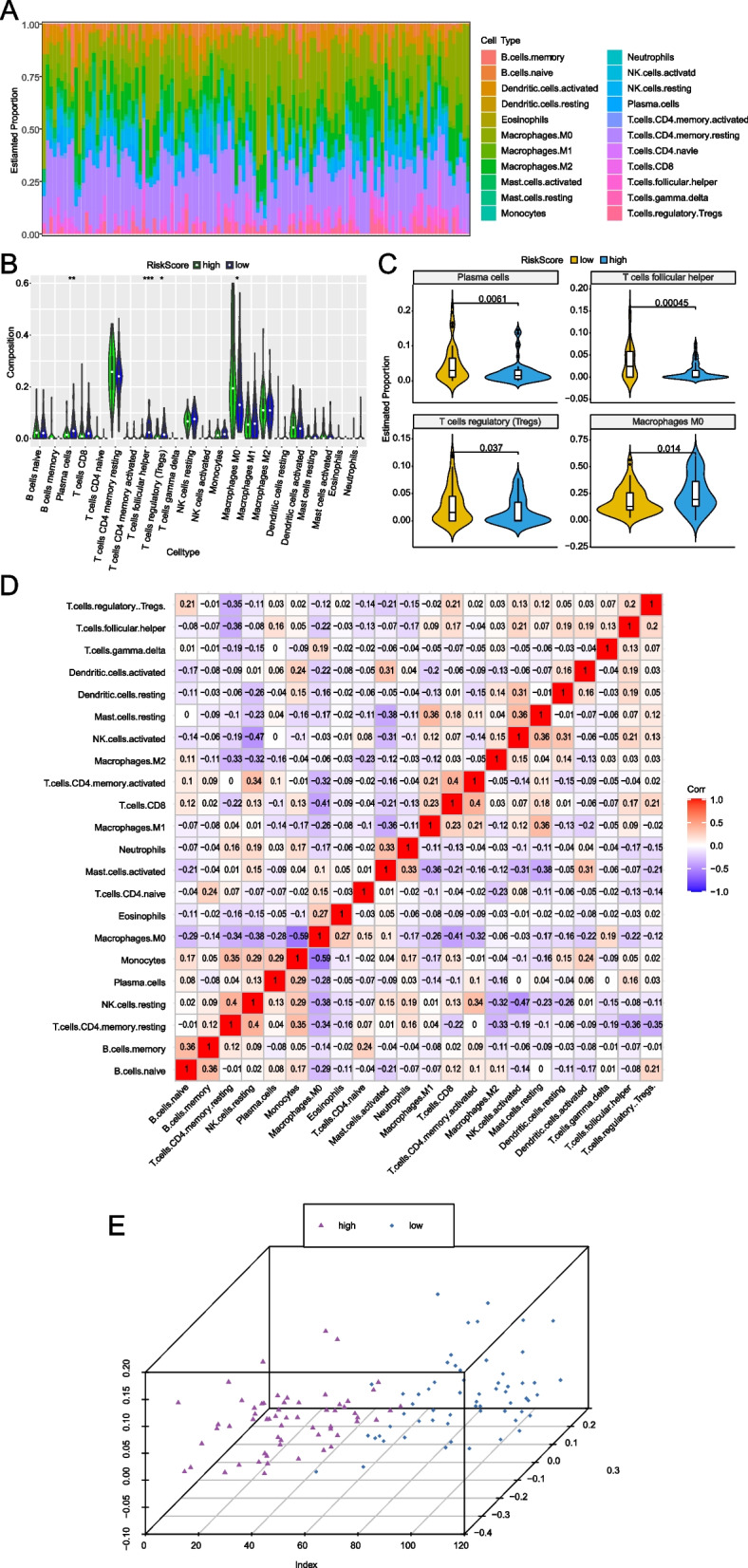
Infiltration ratios of immune cells between the high- and low-risk groups. **A** Estimated proportion of all patients. **B** Box plots of immune cells between high- and low-risk groups. The horizontal axis is the types of 22 immune cells and the vertical is the infiltration ratio of immune cells. *p* value was calculated by the Wilcoxon rank sum test method. *p* > 0.05, *: *p* <  = 0.05, **: *p* <  = 0.01, ***: *p* <  = 0.001, ****: *p* <  = 0.0001. **C**: Box plots of immune cells between high- and low-risk groups. The horizontal axis is the types of 22 immune cells and the vertical is the infiltration ratio of immune cells. *p* value was calculated by the Wilcoxon rank sum test method. **D** Correlation matrix of the proportion of 22 immune cells. The red and blue represent positive and negative correlation. The darker the color, the greater the correlation. **E** The 3-dimensional clustering diagram of PCA. Different colors represent different types of samples

**Fig. 8:**
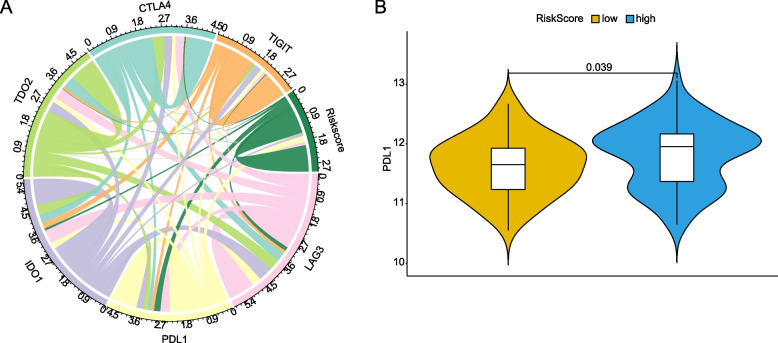
Correlation between immune checkpoints and Risk Score. **A** Chord diagram of Risk Score and 5 immune checkpoints. It was comprised of nodes and lines. The nodes in diagram were Risk Score, TIGIT, CTLA4, TDO2, IDO1, PDL1 and LAG3. Each color represented one node. The width of lines represented the strength of connection between two nodes. **B** Box plots of significantly different expressed immune checkpoints between high- and low-risk groups. The yellow represents the low-risk group and the blue represent high-risk group. The vertical axis is the expression value. *P* value is calculated by the Wilcoxon rank sum test method

After summarizing immune infiltration results of 120 LC patients (Fig. 7A), the variation of infiltration ratio of different patients represented the intrinsic characteristic of individual difference. There were differences in the infiltrating proportion of the immune cells between the high- and low-risk groups (Fig. 7B). And significant differences were observed in the plasma cells, T cells follicular helper, T cells regulatory and macrophage M0. The infiltration ratios of plasma cells, T cells follicular helper and T cells regulatory were higher in the low-risk group, while the infiltration ratio of macrophage M0 was higher in the high-risk group (Fig. 7C). The correlation between the infiltration ratio of different immune cells was weak (Fig. 7D). The 120 LC patients were subjected to clustering analysis based on the infiltration ratio of the four significantly different immune cells. Then the patients were divided into two groups by principal component analysis (PCA), suggesting that the result of infiltration of the immune cells was in accordance with the result of Risk Score (PCA) (Fig. 7E). Moreover, we found that Risk Score was significantly associated with expressions of all of key immune checkpoints (CTLA4, PDL1, LAG3, TIGIT IDO1 and TDO2) (Fig. 8A). Notably, PDL1 showed significantly higher expression in high risk LC patients compared with low risk patients (Fig. 8B).

## Discussion

LC, as the most frequent head and neck cancer in the world, represents a significant source of morbidity and mortality. Although surgery has been the main choice for localized cancer, nonsurgical procedures like radiation and chemotherapy have emerged as an option. In spite of the tremendous improvement in treatment methods over the past few years, LC still is a tumor with poor prognosis when detected at the advanced stage. In order to separate patients with different prognosis, increasing number of studies have shown that the construction of prognostic models based on public databases provides more comprehensive prognostic value. Prognostic models based on TMB-related genes are becoming a research hot topic for predicting prognosis in different cancers.

In this study, we performed GO and KEGG enrichment analyses on the DEGs associated with TMB which were screened by differential analysis. The DEGs were significantly enriched in pathways, such as neuroactive ligand receptor interaction, PI3K-Akt signaling pathway and chemical carcinogenesis receptor activation. DEGs in tongue squamous cell carcinoma also enriched in the PI3K-Akt signaling pathway of KEGG pathways analysis [19]. Previous studies have demonstrated that the tumorigenesis of prostate cancer, including apoptosis and proliferation of cancer cells as well as tumor metastasis and invasion, is correlated with PI3K-Akt signaling pathway. It also showed that the activation of PI3K-Akt mammalian target of rapamycin (PI3K-Akt-mTOR) pathway is of significant importance among abnormal upregulation of leukemogenesis in human acute myeloid leukemia [20]. Therefore targeting key components of PI3K-Akt-mTOR signaling pathway may be an effective therapy method of acute myeloid leukemia. These researches agree with our results of enrichment analysis, suggesting that the DEGs we screened were closely related to cancers.

Six TMB-related genes were screened using univariate Cox and LASSO Cox regression analyses and a Risk Score prognostic model was constructed using the 6 TMB-related genes (FOXJ1, EPO, FGF5, SPOCK1, KCNF1 and PSG5) by multiplying expression of the genes and coefficient from the LASSO Cox analysis. The upregulation genes were FOXJ1 and EPO while the downregulation genes were FGF5, SPOCK1, KCNF1 and PSG5. Overexpression of FOXJ1 enhanced the proliferation and progression of cancer cells of prostate cancer and colorectal cancer [21]. Biomarkers including FOXJ1, CCL22, ABCA3 and IL1RN may be good prognostic factors in breast cancer [22]. EPO gene encodes a secreted glycoprotein hormone. It stimulates growth and prevent apoptosis. Increased expression of EPO has effect on delaying tumor growth could reduce tumor hypoxia and ameliorate the deleterious effects of hypoxia on tumor growth, metastasis and treatment resistance. Previous studies have shown that SPOCK1 may facilitate cancer metastasis in gastric cancer [23]. Knockdown of SPOCK1 expression inhibits the invasion and metastasis of various cancer cells [24]. These studied agree with our results, indicating that the abnormal expression of genes may correlate with the LC. Prognostic models constructed using these genes have shown prognostic values in many types of cancers. Our results showed that the OS of patients in the low-risk group was higher than that in the high-risk group and the death toll of the high-risk group was higher that of the low-risk group. Additionally, although there were much more male LC patients than female cases in TCGA, probably owing to tobacco use habit, our Risk Score still exhibited independent prognostic value in LC patients. Collectively, the Risk Score model we constructed basing on FOXJ1, EPO, FGF5, SPOCK1, KCNF1 and PSG5 displayed more stability, and was a reliable prognostic signature for LC.

The immune infiltration results showed that the infiltration ratios of plasma cells, T cells follicular helper and T cells regulatory were higher in the low-risk group. Plasma cells can secrete antibodies to protect against pathogens. And they showed anti-tumor effect in cancers and had a positive prognostic effect [25]. T cells follicular helper, characterized by the expression of CXC chemokine receptor 5 (CXCR5), are positively connected with survival of cancer patients by the immunoprotective functions of CXCL13 which is correlated with CXCR5 in germinal center [26]. Regulatory T cells play an important role in suppressing inflammation and anti-tumor immune response [27]. Infiltration of large number of regulatory T cells is often associated with poor prognosis [28]. These researches are in accord with our immune infiltration results. Our results of immune cell infiltration is in keeping with Risk Score models, indicating that the Risk Score model is a reliable prognostic model for LC. Furthermore, immune checkpoint expression has become a biomarker for selective immunotherapy in LC patients. The Risk Score herein was associated with the expression of key immune checkpoints, meanwhile PDL1 showed significantly higher expression in high risk LC patients. Upregulation of PDL1 was connected with poor prognosis characteristics, for example metastasis, large tumor size and high proliferation rate and PDL1 mRNA expression may represent an independent prognostic feature in breast cancer. Overexpression of PDL1 was also related to worse outcome of glioblastoma and may be an important indicator for immunotherapy for glioblastoma patients. These agreed with our results that higher expression of PDL1 in the high-risk group indicated poor prognosis of LC patients.

There are some limitations to the present study to be noted. Firstly, the datasets in this study were not large enough to verify the validity of the prognostic Risk Score model and our findings need to be verified in a larger population. Secondly, a multi-center and validation cohort are warranted before exploring clinical significance. And the prognostic signature was developed by six genes, and further experiments are needed to validate its functions in LC.

## Conclusion

The six TMB-related genes were connected with the prognosis of LC. The Risk Score model constructed by six TMB-related genes (FOXJ1, EPO, FGF5, SPOCK1, KCNF1 and PSG5) was a reliable and independent prognostic model for separating LC patients with different prognosis.

## Supplementary Information

**Additional file 1: Table S1.** Full results of enriched GO enrichment analysis of 210 DEGs.

**Additional file 2: Table S2.** Full results of KEGG enrichment analysis of 210 DEGs.

## Data Availability

The datasets used and analyzed during the current study are available in the TCGA database (https://tcga-data.nci.nih.gov/tcga/). The GSE27020 dataset used and analyzed during the current study is available in the Gene Expression Omnibus (GEO, https://www.ncbi.nlm.nih.gov/geo/) database.

## Abbreviations

LC: Laryngeal cancer
TMB: Tumor mutation burden
DEGs: Differentially expressed genes
WES: Whole exome sequencing
TCGA: The Cancer Genome Atlas
GEO: Gene Expression Omnibus
GO: Gene ontology
KEGG: Kyoto Encyclopedia of Genes and Genomes
OS: Overall survival
HR: Hazard ratio
