# Supplementary material for: The prognostic value of tumor mutational burden related 6-gene-based Risk Score in laryngeal cancer patients
Source: BMC Oral Health. 2022 Nov 17;22:510. doi: 10.1186/s12903-022-02534-2 (PMC9673449; doi:10.1186/s12903-022-02534-2)
Supplement: Supplementary file 1 — Additional file 1: Table S1. Full results of enriched GO enrichment analysis of 210 DEGs. [file 12903_2022_2534_MOESM1_ESM.docx]

**Table S1 Full results of enriched GO enrichment analysis of 210 DEGs**

| ONTOLOGY | ID | Description | GeneRatio | BgRatio | pvalue | p.adjust | qvalue | geneID | Count |
| --- | --- | --- | --- | --- | --- | --- | --- | --- | --- |
| BP | GO:0050909 | sensory perception of taste | 6/146 | 66/18862 | 1.20E-05 | 0.025247037 | 0.023473838 | CALHM1/CST2/TAS1R1/CST4/CALHM3/CST1 | 6 |
| BP | GO:0010951 | negative regulation of endopeptidase activity | 9/146 | 245/18862 | 0.000125346 | 0.058539852 | 0.054428367 | CST2/SERPINB7/SERPINA12/SERPINE2/TIMP3/CST4/NGF/SPOCK1/CST1 | 9 |
| BP | GO:0007200 | phospholipase C-activating G protein-coupled receptor signaling pathway | 6/146 | 103/18862 | 0.000149723 | 0.058539852 | 0.054428367 | ADRA1B/F2/CHGA/GRP/LPAR3/ANO1 | 6 |
| BP | GO:0010466 | negative regulation of peptidase activity | 9/146 | 257/18862 | 0.000179518 | 0.058539852 | 0.054428367 | CST2/SERPINB7/SERPINA12/SERPINE2/TIMP3/CST4/NGF/SPOCK1/CST1 | 9 |
| BP | GO:0030198 | extracellular matrix organization | 11/146 | 393/18862 | 0.000247766 | 0.058539852 | 0.054428367 | FOXC2/COL22A1/ADAMTS15/FLRT2/TGFBI/MMP28/MMP7/ADAMTS16/COMP/HPN/MMP13 | 11 |
| BP | GO:0043062 | extracellular structure organization | 11/146 | 394/18862 | 0.000253214 | 0.058539852 | 0.054428367 | FOXC2/COL22A1/ADAMTS15/FLRT2/TGFBI/MMP28/MMP7/ADAMTS16/COMP/HPN/MMP13 | 11 |
| BP | GO:0045229 | external encapsulating structure organization | 11/146 | 396/18862 | 0.000264415 | 0.058539852 | 0.054428367 | FOXC2/COL22A1/ADAMTS15/FLRT2/TGFBI/MMP28/MMP7/ADAMTS16/COMP/HPN/MMP13 | 11 |
| BP | GO:0050913 | sensory perception of bitter taste | 4/146 | 41/18862 | 0.000279235 | 0.058539852 | 0.054428367 | CALHM1/CST2/CST4/CST1 | 4 |
| BP | GO:0010544 | negative regulation of platelet activation | 3/146 | 17/18862 | 0.000285298 | 0.058539852 | 0.054428367 | SERPINE2/THBD/F2 | 3 |
| BP | GO:0042445 | hormone metabolic process | 8/146 | 218/18862 | 0.000301307 | 0.058539852 | 0.054428367 | DIO3/CYP2W1/PCSK4/NR5A1/CYP27C1/NGF/AKR1C2/HPN | 8 |
| BP | GO:0050912 | detection of chemical stimulus involved in sensory perception of taste | 4/146 | 42/18862 | 0.000306783 | 0.058539852 | 0.054428367 | CST2/TAS1R1/CST4/CST1 | 4 |
| BP | GO:0045861 | negative regulation of proteolysis | 10/146 | 346/18862 | 0.000369762 | 0.064677566 | 0.060135005 | CST2/SERPINB7/SERPINA12/SERPINE2/F2/TIMP3/CST4/NGF/SPOCK1/CST1 | 10 |
| BP | GO:0052548 | regulation of endopeptidase activity | 11/146 | 426/18862 | 0.000489726 | 0.079071987 | 0.073518449 | CST2/SERPINB7/SERPINA12/SERPINE2/EGLN3/TIMP3/CST4/NGF/KHDC1L/SPOCK1/CST1 | 11 |
| BP | GO:1900047 | negative regulation of hemostasis | 4/146 | 49/18862 | 0.00055688 | 0.083492251 | 0.077628261 | SERPINE2/THBD/F2/COMP | 4 |
| BP | GO:0048710 | regulation of astrocyte differentiation | 3/146 | 24/18862 | 0.000816139 | 0.103164796 | 0.095919125 | SERPINE2/NOG/F2 | 3 |
| BP | GO:0009581 | detection of external stimulus | 6/146 | 142/18862 | 0.000837575 | 0.103164796 | 0.095919125 | RP1/SERPINE2/CNGB1/CDH2/HPN/ANO1 | 6 |
| BP | GO:0052547 | regulation of peptidase activity | 11/146 | 455/18862 | 0.00084256 | 0.103164796 | 0.095919125 | CST2/SERPINB7/SERPINA12/SERPINE2/EGLN3/TIMP3/CST4/NGF/KHDC1L/SPOCK1/CST1 | 11 |
| BP | GO:0045745 | positive regulation of G protein-coupled receptor signaling pathway | 3/146 | 25/18862 | 0.000922195 | 0.103164796 | 0.095919125 | F2/CHGA/GRP | 3 |
| BP | GO:0009582 | detection of abiotic stimulus | 6/146 | 145/18862 | 0.00093384 | 0.103164796 | 0.095919125 | RP1/SERPINE2/CNGB1/CDH2/HPN/ANO1 | 6 |
| BP | GO:0032835 | glomerulus development | 4/146 | 62/18862 | 0.001356629 | 0.142378193 | 0.13237841 | FOXC2/SERPINB7/NOG/FOXJ1 | 4 |
| BP | GO:0099068 | postsynapse assembly | 3/146 | 30/18862 | 0.001582485 | 0.150279187 | 0.139724486 | SHANK2/CEL/CDH2 | 3 |
| BP | GO:0032963 | collagen metabolic process | 5/146 | 109/18862 | 0.001589695 | 0.150279187 | 0.139724486 | SERPINB7/MMP28/MMP7/F2/MMP13 | 5 |
| BP | GO:0042063 | gliogenesis | 8/146 | 283/18862 | 0.001646699 | 0.150279187 | 0.139724486 | TSPAN2/PLAG1/SERPINE2/NOG/F2/CDH2/FGF5/IL33 | 8 |
| BP | GO:1900046 | regulation of hemostasis | 4/146 | 67/18862 | 0.001809262 | 0.158235041 | 0.14712157 | SERPINE2/THBD/F2/COMP | 4 |
| BP | GO:0048844 | artery morphogenesis | 4/146 | 70/18862 | 0.002125981 | 0.176394784 | 0.164005882 | FOXC2/NOG/FOXH1/COMP | 4 |
| BP | GO:0010543 | regulation of platelet activation | 3/146 | 34/18862 | 0.002280321 | 0.176394784 | 0.164005882 | SERPINE2/THBD/F2 | 3 |
| BP | GO:0048708 | astrocyte differentiation | 4/146 | 72/18862 | 0.002357197 | 0.176394784 | 0.164005882 | TSPAN2/SERPINE2/NOG/F2 | 4 |
| BP | GO:0048672 | positive regulation of collateral sprouting | 2/146 | 10/18862 | 0.002570918 | 0.176394784 | 0.164005882 | NGF/LPAR3 | 2 |
| BP | GO:0048712 | negative regulation of astrocyte differentiation | 2/146 | 10/18862 | 0.002570918 | 0.176394784 | 0.164005882 | NOG/F2 | 2 |
| BP | GO:0051918 | negative regulation of fibrinolysis | 2/146 | 10/18862 | 0.002570918 | 0.176394784 | 0.164005882 | THBD/F2 | 2 |
| BP | GO:0061045 | negative regulation of wound healing | 4/146 | 74/18862 | 0.002605164 | 0.176394784 | 0.164005882 | SERPINE2/THBD/F2/CLDN19 | 4 |
| BP | GO:0001580 | detection of chemical stimulus involved in sensory perception of bitter taste | 3/146 | 36/18862 | 0.002690316 | 0.176467924 | 0.164073886 | CST2/CST4/CST1 | 3 |
| BP | GO:0051346 | negative regulation of hydrolase activity | 10/146 | 456/18862 | 0.002943559 | 0.182281577 | 0.169479223 | CST2/SERPINB7/SERPINA12/SERPINE2/TIMP3/CST4/NGF/SPOCK1/CST1/ELFN2 | 10 |
| BP | GO:0042670 | retinal cone cell differentiation | 2/146 | 11/18862 | 0.003126316 | 0.182281577 | 0.169479223 | DIO3/RP1 | 2 |
| BP | GO:0046549 | retinal cone cell development | 2/146 | 11/18862 | 0.003126316 | 0.182281577 | 0.169479223 | DIO3/RP1 | 2 |
| BP | GO:0097104 | postsynaptic membrane assembly | 2/146 | 11/18862 | 0.003126316 | 0.182281577 | 0.169479223 | CEL/CDH2 | 2 |
| BP | GO:0061041 | regulation of wound healing | 5/146 | 131/18862 | 0.003537583 | 0.200686117 | 0.18659114 | FOXC2/SERPINE2/THBD/F2/CLDN19 | 5 |
| BP | GO:0001886 | endothelial cell morphogenesis | 2/146 | 13/18862 | 0.00438892 | 0.217606723 | 0.202323344 | COL22A1/ARHGEF26 | 2 |
| BP | GO:0010755 | regulation of plasminogen activation | 2/146 | 13/18862 | 0.00438892 | 0.217606723 | 0.202323344 | SERPINE2/HPN | 2 |
| BP | GO:0035845 | photoreceptor cell outer segment organization | 2/146 | 13/18862 | 0.00438892 | 0.217606723 | 0.202323344 | RP1/CNGB1 | 2 |
| BP | GO:0072006 | nephron development | 5/146 | 138/18862 | 0.004414037 | 0.217606723 | 0.202323344 | FOXC2/SERPINB7/NOG/ADAMTS16/FOXJ1 | 5 |
| BP | GO:0001657 | ureteric bud development | 4/146 | 86/18862 | 0.004477204 | 0.217606723 | 0.202323344 | FOXC2/NOG/ADAMTS16/FOXJ1 | 4 |
| BP | GO:0006816 | calcium ion transport | 9/146 | 409/18862 | 0.004621451 | 0.217606723 | 0.202323344 | CALHM1/SLC8A2/JPH2/MRLN/CHRNA4/F2/CACNA1B/EPO/LPAR3 | 9 |
| BP | GO:0072163 | mesonephric epithelium development | 4/146 | 87/18862 | 0.004665223 | 0.217606723 | 0.202323344 | FOXC2/NOG/ADAMTS16/FOXJ1 | 4 |
| BP | GO:0072164 | mesonephric tubule development | 4/146 | 87/18862 | 0.004665223 | 0.217606723 | 0.202323344 | FOXC2/NOG/ADAMTS16/FOXJ1 | 4 |
| BP | GO:0055010 | ventricular cardiac muscle tissue morphogenesis | 3/146 | 44/18862 | 0.004770485 | 0.217679309 | 0.202390832 | FOXC2/NOG/FOXH1 | 3 |
| BP | GO:0051917 | regulation of fibrinolysis | 2/146 | 14/18862 | 0.005094516 | 0.220813546 | 0.205304939 | THBD/F2 | 2 |
| BP | GO:0044706 | multi-multicellular organism process | 6/146 | 204/18862 | 0.005157138 | 0.220813546 | 0.205304939 | SERPINE2/THBD/MMP7/PSG5/PRLR/EPO | 6 |
| BP | GO:0060349 | bone morphogenesis | 4/146 | 90/18862 | 0.005260992 | 0.220813546 | 0.205304939 | RIPPLY2/SFRP4/COMP/MMP13 | 4 |
| BP | GO:1903035 | negative regulation of response to wounding | 4/146 | 90/18862 | 0.005260992 | 0.220813546 | 0.205304939 | SERPINE2/THBD/F2/CLDN19 | 4 |
| BP | GO:0042461 | photoreceptor cell development | 3/146 | 46/18862 | 0.005406717 | 0.220813546 | 0.205304939 | DIO3/RP1/CNGB1 | 3 |
| BP | GO:0001823 | mesonephros development | 4/146 | 91/18862 | 0.005470369 | 0.220813546 | 0.205304939 | FOXC2/NOG/ADAMTS16/FOXJ1 | 4 |
| BP | GO:0030574 | collagen catabolic process | 3/146 | 47/18862 | 0.005743008 | 0.222034415 | 0.206440062 | MMP28/MMP7/MMP13 | 3 |
| BP | GO:0045836 | positive regulation of meiotic nuclear division | 2/146 | 15/18862 | 0.005848584 | 0.222034415 | 0.206440062 | STRA8/PIWIL2 | 2 |
| BP | GO:0014013 | regulation of gliogenesis | 4/146 | 93/18862 | 0.005905686 | 0.222034415 | 0.206440062 | PLAG1/SERPINE2/NOG/F2 | 4 |
| BP | GO:0010001 | glial cell differentiation | 6/146 | 210/18862 | 0.005923739 | 0.222034415 | 0.206440062 | TSPAN2/SERPINE2/NOG/F2/CDH2/FGF5 | 6 |
| BP | GO:0030195 | negative regulation of blood coagulation | 3/146 | 48/18862 | 0.006091576 | 0.224319624 | 0.208564771 | SERPINE2/THBD/F2 | 3 |
| BP | GO:0048705 | skeletal system morphogenesis | 6/146 | 213/18862 | 0.006336786 | 0.225146192 | 0.209333286 | RIPPLY2/FOXC2/NOG/SFRP4/COMP/MMP13 | 6 |
| BP | GO:0060840 | artery development | 4/146 | 95/18862 | 0.0063635 | 0.225146192 | 0.209333286 | FOXC2/NOG/FOXH1/COMP | 4 |
| BP | GO:0050982 | detection of mechanical stimulus | 3/146 | 49/18862 | 0.006452535 | 0.225146192 | 0.209333286 | SERPINE2/CDH2/HPN | 3 |
| BP | GO:0007601 | visual perception | 6/146 | 215/18862 | 0.006623556 | 0.225146192 | 0.209333286 | RP1/TGFBI/POU6F2/TIMP3/CNGB1/CLDN19 | 6 |
| BP | GO:0072109 | glomerular mesangium development | 2/146 | 16/18862 | 0.00665034 | 0.225146192 | 0.209333286 | FOXC2/SERPINB7 | 2 |
| BP | GO:0003229 | ventricular cardiac muscle tissue development | 3/146 | 50/18862 | 0.006825993 | 0.227424738 | 0.211451801 | FOXC2/NOG/FOXH1 | 3 |
| BP | GO:0050953 | sensory perception of light stimulus | 6/146 | 219/18862 | 0.007225229 | 0.236964944 | 0.220321961 | RP1/TGFBI/POU6F2/TIMP3/CNGB1/CLDN19 | 6 |
| BP | GO:0015867 | ATP transport | 2/146 | 17/18862 | 0.007499012 | 0.238434455 | 0.221688262 | CALHM1/CALHM3 | 2 |
| BP | GO:0048670 | regulation of collateral sprouting | 2/146 | 17/18862 | 0.007499012 | 0.238434455 | 0.221688262 | NGF/LPAR3 | 2 |
| BP | GO:0050819 | negative regulation of coagulation | 3/146 | 52/18862 | 0.007610819 | 0.238434455 | 0.221688262 | SERPINE2/THBD/F2 | 3 |
| BP | GO:0030307 | positive regulation of cell growth | 5/146 | 161/18862 | 0.0083774 | 0.258590626 | 0.240428786 | CPNE6/F2/NGF/HPN/LPAR3 | 5 |
| BP | GO:1903034 | regulation of response to wounding | 5/146 | 164/18862 | 0.009031453 | 0.274739423 | 0.255443389 | FOXC2/SERPINE2/THBD/F2/CLDN19 | 5 |
| BP | GO:0072009 | nephron epithelium development | 4/146 | 106/18862 | 0.00930477 | 0.279010183 | 0.259414197 | FOXC2/NOG/ADAMTS16/FOXJ1 | 4 |
| BP | GO:0035265 | organ growth | 5/146 | 166/18862 | 0.009486589 | 0.280455648 | 0.260758141 | FOXC2/PLAG1/NOG/DUSP9/COMP | 5 |
| BP | GO:0055008 | cardiac muscle tissue morphogenesis | 3/146 | 57/18862 | 0.009798346 | 0.285648997 | 0.265586741 | FOXC2/NOG/FOXH1 | 3 |
| BP | GO:0090303 | positive regulation of wound healing | 3/146 | 59/18862 | 0.010765252 | 0.289883478 | 0.269523818 | FOXC2/THBD/F2 | 3 |
| BP | GO:0007416 | synapse assembly | 5/146 | 173/18862 | 0.011203802 | 0.289883478 | 0.269523818 | SHANK2/GPC6/FLRT2/CEL/CDH2 | 5 |
| BP | GO:0046530 | photoreceptor cell differentiation | 3/146 | 60/18862 | 0.01126868 | 0.289883478 | 0.269523818 | DIO3/RP1/CNGB1 | 3 |
| BP | GO:0006590 | thyroid hormone generation | 2/146 | 21/18862 | 0.01134771 | 0.289883478 | 0.269523818 | DIO3/HPN | 2 |
| BP | GO:0072311 | glomerular epithelial cell differentiation | 2/146 | 21/18862 | 0.01134771 | 0.289883478 | 0.269523818 | FOXC2/FOXJ1 | 2 |
| BP | GO:0140013 | meiotic nuclear division | 5/146 | 174/18862 | 0.011465297 | 0.289883478 | 0.269523818 | M1AP/TEX15/SYCP2L/STRA8/PIWIL2 | 5 |
| BP | GO:0008543 | fibroblast growth factor receptor signaling pathway | 4/146 | 113/18862 | 0.011572473 | 0.289883478 | 0.269523818 | FLRT2/NOG/SHISA2/FGF5 | 4 |
| BP | GO:0061448 | connective tissue development | 6/146 | 243/18862 | 0.011692759 | 0.289883478 | 0.269523818 | FOXC2/SERPINB7/PKDCC/TGFBI/NOG/COMP | 6 |
| BP | GO:0043271 | negative regulation of ion transport | 7/146 | 319/18862 | 0.012208714 | 0.289883478 | 0.269523818 | MRLN/PKIA/PKDCC/SERPINE2/CHGA/SFRP4/EPO | 7 |
| BP | GO:0048638 | regulation of developmental growth | 7/146 | 319/18862 | 0.012208714 | 0.289883478 | 0.269523818 | FOXC2/DIO3/CPNE6/NOG/DUSP9/NGF/LPAR3 | 7 |
| BP | GO:0007565 | female pregnancy | 5/146 | 177/18862 | 0.012274697 | 0.289883478 | 0.269523818 | THBD/MMP7/PSG5/PRLR/EPO | 5 |
| BP | GO:0042698 | ovulation cycle | 3/146 | 62/18862 | 0.012315788 | 0.289883478 | 0.269523818 | NR5A1/MMP7/NHLH2 | 3 |
| BP | GO:0045686 | negative regulation of glial cell differentiation | 2/146 | 22/18862 | 0.012419676 | 0.289883478 | 0.269523818 | NOG/F2 | 2 |
| BP | GO:0030001 | metal ion transport | 9/146 | 486/18862 | 0.01347421 | 0.289883478 | 0.269523818 | CALHM1/SLC8A2/JPH2/MRLN/CHRNA4/F2/CACNA1B/EPO/LPAR3 | 9 |
| BP | GO:0015868 | purine ribonucleotide transport | 2/146 | 23/18862 | 0.013534104 | 0.289883478 | 0.269523818 | CALHM1/CALHM3 | 2 |
| BP | GO:0030194 | positive regulation of blood coagulation | 2/146 | 23/18862 | 0.013534104 | 0.289883478 | 0.269523818 | THBD/F2 | 2 |
| BP | GO:0031639 | plasminogen activation | 2/146 | 23/18862 | 0.013534104 | 0.289883478 | 0.269523818 | SERPINE2/HPN | 2 |
| BP | GO:0048668 | collateral sprouting | 2/146 | 23/18862 | 0.013534104 | 0.289883478 | 0.269523818 | NGF/LPAR3 | 2 |
| BP | GO:0051446 | positive regulation of meiotic cell cycle | 2/146 | 23/18862 | 0.013534104 | 0.289883478 | 0.269523818 | STRA8/PIWIL2 | 2 |
| BP | GO:0051503 | adenine nucleotide transport | 2/146 | 23/18862 | 0.013534104 | 0.289883478 | 0.269523818 | CALHM1/CALHM3 | 2 |
| BP | GO:1900048 | positive regulation of hemostasis | 2/146 | 23/18862 | 0.013534104 | 0.289883478 | 0.269523818 | THBD/F2 | 2 |
| BP | GO:0007368 | determination of left/right symmetry | 4/146 | 119/18862 | 0.013776778 | 0.289883478 | 0.269523818 | RIPPLY2/FOXH1/DNAH5/FOXJ1 | 4 |
| BP | GO:0045927 | positive regulation of growth | 6/146 | 253/18862 | 0.014031505 | 0.289883478 | 0.269523818 | DIO3/CPNE6/F2/NGF/HPN/LPAR3 | 6 |
| BP | GO:0030193 | regulation of blood coagulation | 3/146 | 66/18862 | 0.014572468 | 0.289883478 | 0.269523818 | SERPINE2/THBD/F2 | 3 |
| BP | GO:0018230 | peptidyl-L-cysteine S-palmitoylation | 2/146 | 24/18862 | 0.014690283 | 0.289883478 | 0.269523818 | ZDHHC11/ZDHHC11B | 2 |
| BP | GO:0018231 | peptidyl-S-diacylglycerol-L-cysteine biosynthetic process from peptidyl-cysteine | 2/146 | 24/18862 | 0.014690283 | 0.289883478 | 0.269523818 | ZDHHC11/ZDHHC11B | 2 |
| BP | GO:0050820 | positive regulation of coagulation | 2/146 | 24/18862 | 0.014690283 | 0.289883478 | 0.269523818 | THBD/F2 | 2 |
| BP | GO:0051291 | protein heterooligomerization | 2/146 | 24/18862 | 0.014690283 | 0.289883478 | 0.269523818 | CALHM1/CALHM3 | 2 |
| BP | GO:0072010 | glomerular epithelium development | 2/146 | 24/18862 | 0.014690283 | 0.289883478 | 0.269523818 | FOXC2/FOXJ1 | 2 |
| BP | GO:0060415 | muscle tissue morphogenesis | 3/146 | 67/18862 | 0.015170743 | 0.289883478 | 0.269523818 | FOXC2/NOG/FOXH1 | 3 |
| BP | GO:0045685 | regulation of glial cell differentiation | 3/146 | 68/18862 | 0.015782735 | 0.289883478 | 0.269523818 | SERPINE2/NOG/F2 | 3 |
| BP | GO:0051937 | catecholamine transport | 3/146 | 68/18862 | 0.015782735 | 0.289883478 | 0.269523818 | CHRNA4/CHGA/SLC22A3 | 3 |
| BP | GO:0015865 | purine nucleotide transport | 2/146 | 25/18862 | 0.015887508 | 0.289883478 | 0.269523818 | CALHM1/CALHM3 | 2 |
| BP | GO:0042730 | fibrinolysis | 2/146 | 25/18862 | 0.015887508 | 0.289883478 | 0.269523818 | THBD/F2 | 2 |
| BP | GO:0060219 | camera-type eye photoreceptor cell differentiation | 2/146 | 25/18862 | 0.015887508 | 0.289883478 | 0.269523818 | DIO3/RP1 | 2 |
| BP | GO:0072012 | glomerulus vasculature development | 2/146 | 25/18862 | 0.015887508 | 0.289883478 | 0.269523818 | FOXC2/SERPINB7 | 2 |
| BP | GO:1903046 | meiotic cell cycle process | 5/146 | 190/18862 | 0.016230425 | 0.289883478 | 0.269523818 | M1AP/TEX15/SYCP2L/STRA8/PIWIL2 | 5 |
| BP | GO:0003208 | cardiac ventricle morphogenesis | 3/146 | 69/18862 | 0.016408474 | 0.289883478 | 0.269523818 | FOXC2/NOG/FOXH1 | 3 |
| BP | GO:0051966 | regulation of synaptic transmission, glutamatergic | 3/146 | 69/18862 | 0.016408474 | 0.289883478 | 0.269523818 | SHANK2/SERPINE2/CDH2 | 3 |
| BP | GO:0009855 | determination of bilateral symmetry | 4/146 | 126/18862 | 0.016665444 | 0.289883478 | 0.269523818 | RIPPLY2/FOXH1/DNAH5/FOXJ1 | 4 |
| BP | GO:0048015 | phosphatidylinositol-mediated signaling | 5/146 | 192/18862 | 0.016906055 | 0.289883478 | 0.269523818 | SERPINA12/SERPINE2/F2/NGF/EPO | 5 |
| BP | GO:0051960 | regulation of nervous system development | 8/146 | 422/18862 | 0.017086377 | 0.289883478 | 0.269523818 | PLAG1/FLRT2/SERPINE2/NOG/FEZF1/F2/NGF/LPAR3 | 8 |
| BP | GO:0009799 | specification of symmetry | 4/146 | 127/18862 | 0.017106691 | 0.289883478 | 0.269523818 | RIPPLY2/FOXH1/DNAH5/FOXJ1 | 4 |
| BP | GO:0014066 | regulation of phosphatidylinositol 3-kinase signaling | 4/146 | 127/18862 | 0.017106691 | 0.289883478 | 0.269523818 | SERPINA12/SERPINE2/F2/EPO | 4 |
| BP | GO:0006972 | hyperosmotic response | 2/146 | 26/18862 | 0.017125084 | 0.289883478 | 0.269523818 | SST/EPO | 2 |
| BP | GO:0010528 | regulation of transposition | 2/146 | 26/18862 | 0.017125084 | 0.289883478 | 0.269523818 | TEX15/PIWIL2 | 2 |
| BP | GO:0010529 | negative regulation of transposition | 2/146 | 26/18862 | 0.017125084 | 0.289883478 | 0.269523818 | TEX15/PIWIL2 | 2 |
| BP | GO:0042104 | positive regulation of activated T cell proliferation | 2/146 | 26/18862 | 0.017125084 | 0.289883478 | 0.269523818 | HHLA2/EPO | 2 |
| BP | GO:0042403 | thyroid hormone metabolic process | 2/146 | 26/18862 | 0.017125084 | 0.289883478 | 0.269523818 | DIO3/HPN | 2 |
| BP | GO:0061437 | renal system vasculature development | 2/146 | 26/18862 | 0.017125084 | 0.289883478 | 0.269523818 | FOXC2/SERPINB7 | 2 |
| BP | GO:0061440 | kidney vasculature development | 2/146 | 26/18862 | 0.017125084 | 0.289883478 | 0.269523818 | FOXC2/SERPINB7 | 2 |
| BP | GO:0071880 | adenylate cyclase-activating adrenergic receptor signaling pathway | 2/146 | 26/18862 | 0.017125084 | 0.289883478 | 0.269523818 | ADRA1B/CHGA | 2 |
| BP | GO:0050818 | regulation of coagulation | 3/146 | 71/18862 | 0.017701303 | 0.29606884 | 0.275274757 | SERPINE2/THBD/F2 | 3 |
| BP | GO:0051480 | regulation of cytosolic calcium ion concentration | 7/146 | 344/18862 | 0.017772594 | 0.29606884 | 0.275274757 | SLC8A2/JPH2/ADRA1B/F2/CNGB1/EPO/LPAR3 | 7 |
| BP | GO:0048017 | inositol lipid-mediated signaling | 5/146 | 196/18862 | 0.018312861 | 0.296183077 | 0.275380971 | SERPINA12/SERPINE2/F2/NGF/EPO | 5 |
| BP | GO:1903036 | positive regulation of response to wounding | 3/146 | 72/18862 | 0.018368438 | 0.296183077 | 0.275380971 | FOXC2/THBD/F2 | 3 |
| BP | GO:0032967 | positive regulation of collagen biosynthetic process | 2/146 | 27/18862 | 0.018402323 | 0.296183077 | 0.275380971 | SERPINB7/F2 | 2 |
| BP | GO:0035264 | multicellular organism growth | 4/146 | 130/18862 | 0.018474062 | 0.296183077 | 0.275380971 | PLAG1/DIO3/PKDCC/COMP | 4 |
| BP | GO:0050767 | regulation of neurogenesis | 7/146 | 348/18862 | 0.018805271 | 0.296183077 | 0.275380971 | PLAG1/SERPINE2/NOG/FEZF1/F2/NGF/LPAR3 | 7 |
| BP | GO:0007292 | female gamete generation | 4/146 | 131/18862 | 0.018944502 | 0.296183077 | 0.275380971 | NOBOX/M1AP/STRA8/PIWIL2 | 4 |
| BP | GO:0072073 | kidney epithelium development | 4/146 | 131/18862 | 0.018944502 | 0.296183077 | 0.275380971 | FOXC2/NOG/ADAMTS16/FOXJ1 | 4 |
| BP | GO:0003151 | outflow tract morphogenesis | 3/146 | 73/18862 | 0.019049412 | 0.296183077 | 0.275380971 | FOXC2/NOG/FOXH1 | 3 |
| BP | GO:0048644 | muscle organ morphogenesis | 3/146 | 73/18862 | 0.019049412 | 0.296183077 | 0.275380971 | FOXC2/NOG/FOXH1 | 3 |
| BP | GO:0010714 | positive regulation of collagen metabolic process | 2/146 | 28/18862 | 0.019718547 | 0.304332568 | 0.282958091 | SERPINB7/F2 | 2 |
| BP | GO:0009952 | anterior/posterior pattern specification | 5/146 | 203/18862 | 0.020956833 | 0.31874601 | 0.29635922 | RIPPLY2/FOXC2/NOG/FEZF1/FOXH1 | 5 |
| BP | GO:0097106 | postsynaptic density organization | 2/146 | 29/18862 | 0.021073083 | 0.31874601 | 0.29635922 | SHANK2/CDH2 | 2 |
| BP | GO:0035082 | axoneme assembly | 3/146 | 76/18862 | 0.021175499 | 0.31874601 | 0.29635922 | RP1/DNAH5/FOXJ1 | 3 |
| BP | GO:0022412 | cellular process involved in reproduction in multicellular organism | 7/146 | 357/18862 | 0.021281785 | 0.31874601 | 0.29635922 | NOBOX/M1AP/TEX15/PCSK4/STRA8/SOX30/PIWIL2 | 7 |
| BP | GO:0006874 | cellular calcium ion homeostasis | 8/146 | 442/18862 | 0.021857983 | 0.31874601 | 0.29635922 | SLC8A2/JPH2/ADRA1B/F2/CNGB1/EPO/LPAR3/ATP13A5 | 8 |
| BP | GO:0061418 | regulation of transcription from RNA polymerase II promoter in response to hypoxia | 3/146 | 77/18862 | 0.021911946 | 0.31874601 | 0.29635922 | EGLN3/HIF3A/EPO | 3 |
| BP | GO:1901616 | organic hydroxy compound catabolic process | 3/146 | 77/18862 | 0.021911946 | 0.31874601 | 0.29635922 | MIOX/DIO3/CEL | 3 |
| BP | GO:0002920 | regulation of humoral immune response | 4/146 | 138/18862 | 0.02244569 | 0.31874601 | 0.29635922 | CFHR4/F2/C7/FOXJ1 | 4 |
| BP | GO:0007586 | digestion | 4/146 | 138/18862 | 0.02244569 | 0.31874601 | 0.29635922 | LCT/SST/CEL/AKR1C2 | 4 |
| BP | GO:0061384 | heart trabecula morphogenesis | 2/146 | 30/18862 | 0.022465268 | 0.31874601 | 0.29635922 | NOG/FOXH1 | 2 |
| BP | GO:0071711 | basement membrane organization | 2/146 | 30/18862 | 0.022465268 | 0.31874601 | 0.29635922 | FLRT2/HPN | 2 |
| BP | GO:0002064 | epithelial cell development | 5/146 | 207/18862 | 0.0225741 | 0.31874601 | 0.29635922 | FOXC2/COL22A1/ARHGEF26/CDH2/FOXJ1 | 5 |
| BP | GO:0015844 | monoamine transport | 3/146 | 79/18862 | 0.023426488 | 0.31874601 | 0.29635922 | CHRNA4/CHGA/SLC22A3 | 3 |
| BP | GO:0030111 | regulation of Wnt signaling pathway | 7/146 | 365/18862 | 0.023666587 | 0.31874601 | 0.29635922 | FOXL1/ATP6V1C2/NOG/SHISA2/SFRP4/CDH2/SOX30 | 7 |
| BP | GO:0003382 | epithelial cell morphogenesis | 2/146 | 31/18862 | 0.023894448 | 0.31874601 | 0.29635922 | COL22A1/ARHGEF26 | 2 |
| BP | GO:0010259 | multicellular organism aging | 2/146 | 31/18862 | 0.023894448 | 0.31874601 | 0.29635922 | NR5A1/COMP | 2 |
| BP | GO:0019835 | cytolysis | 2/146 | 31/18862 | 0.023894448 | 0.31874601 | 0.29635922 | F2/C7 | 2 |
| BP | GO:0032196 | transposition | 2/146 | 31/18862 | 0.023894448 | 0.31874601 | 0.29635922 | TEX15/PIWIL2 | 2 |
| BP | GO:0040020 | regulation of meiotic nuclear division | 2/146 | 31/18862 | 0.023894448 | 0.31874601 | 0.29635922 | STRA8/PIWIL2 | 2 |
| BP | GO:0071875 | adrenergic receptor signaling pathway | 2/146 | 31/18862 | 0.023894448 | 0.31874601 | 0.29635922 | ADRA1B/CHGA | 2 |
| BP | GO:0099084 | postsynaptic specialization organization | 2/146 | 31/18862 | 0.023894448 | 0.31874601 | 0.29635922 | SHANK2/CDH2 | 2 |
| BP | GO:0001895 | retina homeostasis | 3/146 | 80/18862 | 0.024204581 | 0.31874601 | 0.29635922 | RP1/CST4/CNGB1 | 3 |
| BP | GO:2000243 | positive regulation of reproductive process | 3/146 | 80/18862 | 0.024204581 | 0.31874601 | 0.29635922 | NR5A1/STRA8/PIWIL2 | 3 |
| BP | GO:0044344 | cellular response to fibroblast growth factor stimulus | 4/146 | 142/18862 | 0.024612172 | 0.31874601 | 0.29635922 | FLRT2/NOG/SHISA2/FGF5 | 4 |
| BP | GO:0030178 | negative regulation of Wnt signaling pathway | 5/146 | 212/18862 | 0.024706942 | 0.31874601 | 0.29635922 | NOG/SHISA2/SFRP4/CDH2/SOX30 | 5 |
| BP | GO:0022617 | extracellular matrix disassembly | 3/146 | 81/18862 | 0.02499655 | 0.31874601 | 0.29635922 | MMP7/HPN/MMP13 | 3 |
| BP | GO:0055074 | calcium ion homeostasis | 8/146 | 454/18862 | 0.025137536 | 0.31874601 | 0.29635922 | SLC8A2/JPH2/ADRA1B/F2/CNGB1/EPO/LPAR3/ATP13A5 | 8 |
| BP | GO:0006862 | nucleotide transport | 2/146 | 32/18862 | 0.025359973 | 0.31874601 | 0.29635922 | CALHM1/CALHM3 | 2 |
| BP | GO:0016486 | peptide hormone processing | 2/146 | 32/18862 | 0.025359973 | 0.31874601 | 0.29635922 | PCSK4/NGF | 2 |
| BP | GO:0042462 | eye photoreceptor cell development | 2/146 | 32/18862 | 0.025359973 | 0.31874601 | 0.29635922 | DIO3/RP1 | 2 |
| BP | GO:0050974 | detection of mechanical stimulus involved in sensory perception | 2/146 | 32/18862 | 0.025359973 | 0.31874601 | 0.29635922 | SERPINE2/HPN | 2 |
| BP | GO:0008277 | regulation of G protein-coupled receptor signaling pathway | 4/146 | 144/18862 | 0.025741231 | 0.321612163 | 0.299024072 | F2/CHGA/CNGB1/GRP | 4 |
| BP | GO:0060291 | long-term synaptic potentiation | 3/146 | 83/18862 | 0.026622077 | 0.3259056 | 0.303015963 | SLC8A2/SHANK2/SERPINE2 | 3 |
| BP | GO:0070509 | calcium ion import | 3/146 | 83/18862 | 0.026622077 | 0.3259056 | 0.303015963 | SLC8A2/MRLN/CACNA1B | 3 |
| BP | GO:0018345 | protein palmitoylation | 2/146 | 33/18862 | 0.026861205 | 0.3259056 | 0.303015963 | ZDHHC11/ZDHHC11B | 2 |
| BP | GO:0042573 | retinoic acid metabolic process | 2/146 | 33/18862 | 0.026861205 | 0.3259056 | 0.303015963 | CYP2W1/CYP27C1 | 2 |
| BP | GO:0140448 | signaling receptor ligand precursor processing | 2/146 | 33/18862 | 0.026861205 | 0.3259056 | 0.303015963 | PCSK4/NGF | 2 |
| BP | GO:0061053 | somite development | 3/146 | 84/18862 | 0.027455612 | 0.331203044 | 0.307941347 | RIPPLY2/FOXC2/NOG | 3 |
| BP | GO:0071774 | response to fibroblast growth factor | 4/146 | 148/18862 | 0.02809176 | 0.33694059 | 0.313275923 | FLRT2/NOG/SHISA2/FGF5 | 4 |
| BP | GO:0048477 | oogenesis | 3/146 | 85/18862 | 0.028302975 | 0.337545137 | 0.31383801 | NOBOX/STRA8/PIWIL2 | 3 |
| BP | GO:0007603 | phototransduction, visible light | 2/146 | 35/18862 | 0.029968264 | 0.348354085 | 0.323887803 | RP1/CNGB1 | 2 |
| BP | GO:0033363 | secretory granule organization | 2/146 | 35/18862 | 0.029968264 | 0.348354085 | 0.323887803 | SERPINE2/SOX30 | 2 |
| BP | GO:0040036 | regulation of fibroblast growth factor receptor signaling pathway | 2/146 | 35/18862 | 0.029968264 | 0.348354085 | 0.323887803 | NOG/SHISA2 | 2 |
| BP | GO:0048854 | brain morphogenesis | 2/146 | 35/18862 | 0.029968264 | 0.348354085 | 0.323887803 | SHANK2/CDH2 | 2 |
| BP | GO:0006919 | activation of cysteine-type endopeptidase activity involved in apoptotic process | 3/146 | 87/18862 | 0.030039109 | 0.348354085 | 0.323887803 | EGLN3/NGF/KHDC1L | 3 |
| BP | GO:0016485 | protein processing | 5/146 | 224/18862 | 0.030342853 | 0.349943126 | 0.325365239 | PCSK4/SERPINE2/NGF/COMP/HPN | 5 |
| BP | GO:0060402 | calcium ion transport into cytosol | 4/146 | 152/18862 | 0.030566381 | 0.350594725 | 0.325971074 | SLC8A2/JPH2/F2/EPO | 4 |
| BP | GO:0060993 | kidney morphogenesis | 3/146 | 88/18862 | 0.030927837 | 0.35281266 | 0.328033235 | NOG/ADAMTS16/FOXJ1 | 3 |
| BP | GO:0070588 | calcium ion transmembrane transport | 6/146 | 304/18862 | 0.031121478 | 0.353102607 | 0.328302817 | CALHM1/SLC8A2/JPH2/MRLN/F2/CACNA1B | 6 |
| BP | GO:0014014 | negative regulation of gliogenesis | 2/146 | 36/18862 | 0.031572849 | 0.354392568 | 0.329502179 | NOG/F2 | 2 |
| BP | GO:0071709 | membrane assembly | 2/146 | 36/18862 | 0.031572849 | 0.354392568 | 0.329502179 | CEL/CDH2 | 2 |
| BP | GO:0014065 | phosphatidylinositol 3-kinase signaling | 4/146 | 154/18862 | 0.031850507 | 0.35560752 | 0.330631801 | SERPINA12/SERPINE2/F2/EPO | 4 |
| BP | GO:0014068 | positive regulation of phosphatidylinositol 3-kinase signaling | 3/146 | 90/18862 | 0.032746489 | 0.361762532 | 0.336354522 | SERPINA12/F2/EPO | 3 |
| BP | GO:0035249 | synaptic transmission, glutamatergic | 3/146 | 90/18862 | 0.032746489 | 0.361762532 | 0.336354522 | SHANK2/SERPINE2/CDH2 | 3 |
| BP | GO:0001941 | postsynaptic membrane organization | 2/146 | 37/18862 | 0.033210655 | 0.364969453 | 0.339336209 | CEL/CDH2 | 2 |
| BP | GO:0072503 | cellular divalent inorganic cation homeostasis | 8/146 | 480/18862 | 0.033397249 | 0.365108462 | 0.339465454 | SLC8A2/JPH2/ADRA1B/F2/CNGB1/EPO/LPAR3/ATP13A5 | 8 |
| BP | GO:0007204 | positive regulation of cytosolic calcium ion concentration | 6/146 | 310/18862 | 0.033757447 | 0.366085809 | 0.340374158 | SLC8A2/JPH2/ADRA1B/F2/EPO/LPAR3 | 6 |
| BP | GO:0030168 | platelet activation | 4/146 | 157/18862 | 0.033835468 | 0.366085809 | 0.340374158 | SERPINE2/THBD/F2/COMP | 4 |
| BP | GO:0007618 | mating | 2/146 | 38/18862 | 0.03488108 | 0.373547893 | 0.34731215 | SERPINE2/NHLH2 | 2 |
| BP | GO:0045823 | positive regulation of heart contraction | 2/146 | 38/18862 | 0.03488108 | 0.373547893 | 0.34731215 | ADRA1B/CHGA | 2 |
| BP | GO:0060284 | regulation of cell development | 8/146 | 485/18862 | 0.035175375 | 0.374787375 | 0.348464579 | PLAG1/DIO3/SERPINE2/NOG/FEZF1/F2/NGF/LPAR3 | 8 |
| BP | GO:0001501 | skeletal system development | 8/146 | 486/18862 | 0.035538575 | 0.37674479 | 0.350284517 | RIPPLY2/FOXC2/PKDCC/TGFBI/NOG/SFRP4/COMP/MMP13 | 8 |
| BP | GO:0045666 | positive regulation of neuron differentiation | 3/146 | 94/18862 | 0.03654774 | 0.383568536 | 0.356629004 | FEZF1/NGF/EPO | 3 |
| BP | GO:0070167 | regulation of biomineral tissue development | 3/146 | 94/18862 | 0.03654774 | 0.383568536 | 0.356629004 | PKDCC/COMP/AMTN | 3 |
| BP | GO:0010522 | regulation of calcium ion transport into cytosol | 3/146 | 95/18862 | 0.037532009 | 0.387855342 | 0.36061473 | JPH2/F2/EPO | 3 |
| BP | GO:0051924 | regulation of calcium ion transport | 5/146 | 238/18862 | 0.037868646 | 0.387855342 | 0.36061473 | JPH2/MRLN/F2/EPO/LPAR3 | 5 |
| BP | GO:0010719 | negative regulation of epithelial to mesenchymal transition | 2/146 | 40/18862 | 0.038317409 | 0.387855342 | 0.36061473 | NOG/HPN | 2 |
| BP | GO:0051281 | positive regulation of release of sequestered calcium ion into cytosol | 2/146 | 40/18862 | 0.038317409 | 0.387855342 | 0.36061473 | JPH2/F2 | 2 |
| BP | GO:1903524 | positive regulation of blood circulation | 2/146 | 40/18862 | 0.038317409 | 0.387855342 | 0.36061473 | ADRA1B/CHGA | 2 |
| BP | GO:0050804 | modulation of chemical synaptic transmission | 7/146 | 405/18862 | 0.038373784 | 0.387855342 | 0.36061473 | SLC8A2/SHANK2/SERPINE2/CEL/NGF/CACNA1B/CDH2 | 7 |
| BP | GO:0001655 | urogenital system development | 6/146 | 320/18862 | 0.038465093 | 0.387855342 | 0.36061473 | FOXC2/PLAG1/SERPINB7/NOG/ADAMTS16/FOXJ1 | 6 |
| BP | GO:0110149 | regulation of biomineralization | 3/146 | 96/18862 | 0.038529784 | 0.387855342 | 0.36061473 | PKDCC/COMP/AMTN | 3 |
| BP | GO:0001558 | regulation of cell growth | 7/146 | 406/18862 | 0.038804012 | 0.387855342 | 0.36061473 | SERPINE2/CPNE6/F2/NGF/HPN/SPOCK1/LPAR3 | 7 |
| BP | GO:0099177 | regulation of trans-synaptic signaling | 7/146 | 406/18862 | 0.038804012 | 0.387855342 | 0.36061473 | SLC8A2/SHANK2/SERPINE2/CEL/NGF/CACNA1B/CDH2 | 7 |
| BP | GO:0030850 | prostate gland development | 2/146 | 41/18862 | 0.040082145 | 0.396851044 | 0.368978629 | PLAG1/NOG | 2 |
| BP | GO:0044091 | membrane biogenesis | 2/146 | 41/18862 | 0.040082145 | 0.396851044 | 0.368978629 | CEL/CDH2 | 2 |
| BP | GO:0072507 | divalent inorganic cation homeostasis | 8/146 | 499/18862 | 0.04049366 | 0.399043153 | 0.371016778 | SLC8A2/JPH2/ADRA1B/F2/CNGB1/EPO/LPAR3/ATP13A5 | 8 |
| BP | GO:0048736 | appendage development | 4/146 | 167/18862 | 0.040963821 | 0.399140377 | 0.371107173 | PKDCC/NOG/COMP/KRT84 | 4 |
| BP | GO:0060173 | limb development | 4/146 | 167/18862 | 0.040963821 | 0.399140377 | 0.371107173 | PKDCC/NOG/COMP/KRT84 | 4 |
| BP | GO:0090596 | sensory organ morphogenesis | 5/146 | 244/18862 | 0.041414824 | 0.399140377 | 0.371107173 | DIO3/RP1/TENM3/NOG/HPN | 5 |
| BP | GO:0003002 | regionalization | 6/146 | 326/18862 | 0.041481502 | 0.399140377 | 0.371107173 | RIPPLY2/FOXC2/NOG/FEZF1/FOXH1/FOXJ1 | 6 |
| BP | GO:0006509 | membrane protein ectodomain proteolysis | 2/146 | 42/18862 | 0.041877158 | 0.399140377 | 0.371107173 | MMP7/TIMP3 | 2 |
| BP | GO:0032965 | regulation of collagen biosynthetic process | 2/146 | 42/18862 | 0.041877158 | 0.399140377 | 0.371107173 | SERPINB7/F2 | 2 |
| BP | GO:0072595 | maintenance of protein localization in organelle | 2/146 | 42/18862 | 0.041877158 | 0.399140377 | 0.371107173 | PDIA2/NR5A1 | 2 |
| BP | GO:0003018 | vascular process in circulatory system | 5/146 | 245/18862 | 0.042024785 | 0.399140377 | 0.371107173 | FOXC2/SLC8A2/ADRA1B/COMP/SLC22A3 | 5 |
| BP | GO:0060079 | excitatory postsynaptic potential | 3/146 | 100/18862 | 0.042655081 | 0.401493341 | 0.373294879 | SLC8A2/CHRNA4/SHANK2 | 3 |
| BP | GO:0060419 | heart growth | 3/146 | 100/18862 | 0.042655081 | 0.401493341 | 0.373294879 | FOXC2/NOG/DUSP9 | 3 |
| BP | GO:0048639 | positive regulation of developmental growth | 4/146 | 170/18862 | 0.043256293 | 0.402325674 | 0.374068754 | DIO3/CPNE6/NGF/LPAR3 | 4 |
| BP | GO:0010959 | regulation of metal ion transport | 5/146 | 247/18862 | 0.043260996 | 0.402325674 | 0.374068754 | JPH2/MRLN/F2/EPO/LPAR3 | 5 |
| BP | GO:0001754 | eye photoreceptor cell differentiation | 2/146 | 43/18862 | 0.043701884 | 0.402325674 | 0.374068754 | DIO3/RP1 | 2 |
| BP | GO:0046006 | regulation of activated T cell proliferation | 2/146 | 43/18862 | 0.043701884 | 0.402325674 | 0.374068754 | HHLA2/EPO | 2 |
| BP | GO:0061383 | trabecula morphogenesis | 2/146 | 43/18862 | 0.043701884 | 0.402325674 | 0.374068754 | NOG/FOXH1 | 2 |
| BP | GO:0046620 | regulation of organ growth | 3/146 | 102/18862 | 0.044797596 | 0.407472621 | 0.378854211 | FOXC2/NOG/DUSP9 | 3 |
| BP | GO:0051321 | meiotic cell cycle | 5/146 | 250/18862 | 0.045156121 | 0.407472621 | 0.378854211 | M1AP/TEX15/SYCP2L/STRA8/PIWIL2 | 5 |
| BP | GO:0007339 | binding of sperm to zona pellucida | 2/146 | 44/18862 | 0.04555576 | 0.407472621 | 0.378854211 | PCSK4/ZAN | 2 |
| BP | GO:0045494 | photoreceptor cell maintenance | 2/146 | 44/18862 | 0.04555576 | 0.407472621 | 0.378854211 | RP1/CNGB1 | 2 |
| BP | GO:0046427 | positive regulation of receptor signaling pathway via JAK-STAT | 2/146 | 44/18862 | 0.04555576 | 0.407472621 | 0.378854211 | F2/PRLR | 2 |
| BP | GO:0098815 | modulation of excitatory postsynaptic potential | 2/146 | 44/18862 | 0.04555576 | 0.407472621 | 0.378854211 | SLC8A2/SHANK2 | 2 |
| BP | GO:0060401 | cytosolic calcium ion transport | 4/146 | 173/18862 | 0.04561985 | 0.407472621 | 0.378854211 | SLC8A2/JPH2/F2/EPO | 4 |
| BP | GO:0002062 | chondrocyte differentiation | 3/146 | 103/18862 | 0.045888646 | 0.408136725 | 0.379471672 | PKDCC/TGFBI/COMP | 3 |
| BP | GO:0007389 | pattern specification process | 7/146 | 426/18862 | 0.048081825 | 0.422787917 | 0.393093854 | RIPPLY2/FOXC2/NOG/FEZF1/FOXH1/DNAH5/FOXJ1 | 7 |
| BP | GO:0099565 | chemical synaptic transmission, postsynaptic | 3/146 | 105/18862 | 0.048110058 | 0.422787917 | 0.393093854 | SLC8A2/CHRNA4/SHANK2 | 3 |
| BP | GO:0008217 | regulation of blood pressure | 4/146 | 177/18862 | 0.048881688 | 0.422787917 | 0.393093854 | ADRA1B/CHGA/TRHDE/ADAMTS16 | 4 |
| BP | GO:0018958 | phenol-containing compound metabolic process | 3/146 | 106/18862 | 0.049240309 | 0.422787917 | 0.393093854 | DIO3/PNMT/HPN | 3 |
| BP | GO:0002686 | negative regulation of leukocyte migration | 2/146 | 46/18862 | 0.049348756 | 0.422787917 | 0.393093854 | MMP28/IL33 | 2 |
| BP | GO:0006953 | acute-phase response | 2/146 | 46/18862 | 0.049348756 | 0.422787917 | 0.393093854 | F2/EPO | 2 |
| BP | GO:0007140 | male meiotic nuclear division | 2/146 | 46/18862 | 0.049348756 | 0.422787917 | 0.393093854 | M1AP/TEX15 | 2 |
| BP | GO:0050798 | activated T cell proliferation | 2/146 | 46/18862 | 0.049348756 | 0.422787917 | 0.393093854 | HHLA2/EPO | 2 |
| BP | GO:0097720 | calcineurin-mediated signaling | 2/146 | 46/18862 | 0.049348756 | 0.422787917 | 0.393093854 | SLC8A2/NR5A1 | 2 |
| CC | GO:0062023 | collagen-containing extracellular matrix | 13/158 | 423/19520 | 4.17E-05 | 0.008628806 | 0.008161495 | GPC6/ADAMTS15/TGFBI/SERPINE2/MMP28/F2/TIMP3/LMAN1L/CDH2/COMP/PRG4/THBS4/AMTN | 13 |
| CC | GO:0005788 | endoplasmic reticulum lumen | 8/158 | 306/19520 | 0.003527784 | 0.264922043 | 0.250574625 | PDIA2/COL22A1/CYP2W1/F2/CES3/ARSI/CDH2/AMTN | 8 |
| CC | GO:0016528 | sarcoplasm | 4/158 | 79/19520 | 0.003877475 | 0.264922043 | 0.250574625 | JPH2/MRLN/SPOCK1/THBS4 | 4 |
| CC | GO:0010369 | chromocenter | 2/158 | 15/19520 | 0.006379109 | 0.264922043 | 0.250574625 | SOX30/PIWIL2 | 2 |
| CC | GO:0098793 | presynapse | 10/158 | 487/19520 | 0.006399083 | 0.264922043 | 0.250574625 | SLC8A2/SV2B/LGI3/CEL/CNGB1/NGF/DOC2A/CDH2/NTS/SLC22A3 | 10 |
| CC | GO:0016327 | apicolateral plasma membrane | 2/158 | 21/19520 | 0.01235965 | 0.399878235 | 0.378221977 | THBD/CDH2 | 2 |
| CC | GO:0097733 | photoreceptor cell cilium | 4/158 | 115/19520 | 0.01427689 | 0.399878235 | 0.378221977 | SHANK2/RP1/TSGA10IP/CNGB1 | 4 |
| CC | GO:0030133 | transport vesicle | 8/158 | 402/19520 | 0.016808222 | 0.399878235 | 0.378221977 | SV2B/CHGA/LGI3/NGF/DOC2A/NTS/SFTA2/IL33 | 8 |
| CC | GO:0016529 | sarcoplasmic reticulum | 3/158 | 70/19520 | 0.019183079 | 0.399878235 | 0.378221977 | JPH2/MRLN/THBS4 | 3 |
| CC | GO:0097731 | 9+0 non-motile cilium | 4/158 | 126/19520 | 0.019317789 | 0.399878235 | 0.378221977 | SHANK2/RP1/TSGA10IP/CNGB1 | 4 |
| CC | GO:0016324 | apical plasma membrane | 7/158 | 351/19520 | 0.024379988 | 0.458787042 | 0.433940452 | SHANK2/LCT/ANXA13/TMEM235/CDH2/HPN/ANO1 | 7 |
| CC | GO:0016323 | basolateral plasma membrane | 5/158 | 211/19520 | 0.028732016 | 0.465544992 | 0.44033241 | CALHM1/SLC8A2/CALHM3/CLDN19/CDH2 | 5 |
| CC | GO:0031904 | endosome lumen | 2/158 | 35/19520 | 0.032535142 | 0.465544992 | 0.44033241 | NGF/PRLR | 2 |
| CC | GO:0001750 | photoreceptor outer segment | 3/158 | 89/19520 | 0.035644458 | 0.465544992 | 0.44033241 | SHANK2/RP1/CNGB1 | 3 |
| CC | GO:0034702 | ion channel complex | 6/158 | 301/19520 | 0.036022273 | 0.465544992 | 0.44033241 | CHRNA4/SHANK2/CNGB1/KCNF1/CACNA1B/ANO1 | 6 |
| CC | GO:0032391 | photoreceptor connecting cilium | 2/158 | 39/19520 | 0.03968075 | 0.465544992 | 0.44033241 | RP1/TSGA10IP | 2 |
| CC | GO:0005604 | basement membrane | 3/158 | 94/19520 | 0.040875877 | 0.465544992 | 0.44033241 | TGFBI/THBS4/AMTN | 3 |
| CC | GO:0097730 | non-motile cilium | 4/158 | 160/19520 | 0.041227543 | 0.465544992 | 0.44033241 | SHANK2/RP1/TSGA10IP/CNGB1 | 4 |
| CC | GO:0033017 | sarcoplasmic reticulum membrane | 2/158 | 41/19520 | 0.043455794 | 0.465544992 | 0.44033241 | JPH2/MRLN | 2 |
| CC | GO:0009925 | basal plasma membrane | 5/158 | 240/19520 | 0.045838023 | 0.465544992 | 0.44033241 | CALHM1/SLC8A2/CALHM3/CLDN19/CDH2 | 5 |
| CC | GO:1902495 | transmembrane transporter complex | 6/158 | 323/19520 | 0.047911803 | 0.465544992 | 0.44033241 | CHRNA4/SHANK2/CNGB1/KCNF1/CACNA1B/ANO1 | 6 |
| MF | GO:0004866 | endopeptidase inhibitor activity | 9/149 | 180/18337 | 1.66E-05 | 0.003271869 | 0.002967351 | CST2/SERPINB7/SERPINA12/SERPINE2/TIMP3/CST4/NGF/SPOCK1/CST1 | 9 |
| MF | GO:0030414 | peptidase inhibitor activity | 9/149 | 187/18337 | 2.25E-05 | 0.003271869 | 0.002967351 | CST2/SERPINB7/SERPINA12/SERPINE2/TIMP3/CST4/NGF/SPOCK1/CST1 | 9 |
| MF | GO:0061135 | endopeptidase regulator activity | 9/149 | 192/18337 | 2.77E-05 | 0.003271869 | 0.002967351 | CST2/SERPINB7/SERPINA12/SERPINE2/TIMP3/CST4/NGF/SPOCK1/CST1 | 9 |
| MF | GO:0004857 | enzyme inhibitor activity | 12/149 | 385/18337 | 7.41E-05 | 0.006558072 | 0.005947701 | MRLN/CST2/SERPINB7/PKIA/SERPINA12/SERPINE2/TIMP3/CST4/NGF/SPOCK1/CST1/ELFN2 | 12 |
| MF | GO:0061134 | peptidase regulator activity | 9/149 | 230/18337 | 0.000112053 | 0.007933367 | 0.007194995 | CST2/SERPINB7/SERPINA12/SERPINE2/TIMP3/CST4/NGF/SPOCK1/CST1 | 9 |
| MF | GO:0008191 | metalloendopeptidase inhibitor activity | 3/149 | 16/18337 | 0.000272453 | 0.016074735 | 0.014578633 | TIMP3/NGF/SPOCK1 | 3 |
| MF | GO:0008201 | heparin binding | 7/149 | 164/18337 | 0.000390132 | 0.019729523 | 0.017893264 | ADAMTS15/SERPINE2/MMP7/F2/CEL/COMP/THBS4 | 7 |
| MF | GO:0008237 | metallopeptidase activity | 7/149 | 184/18337 | 0.00077424 | 0.034260139 | 0.031071491 | ADAMTS15/MMP28/MMP7/TRHDE/ADAMTS16/CPA2/MMP13 | 7 |
| MF | GO:0004869 | cysteine-type endopeptidase inhibitor activity | 4/149 | 56/18337 | 0.001107749 | 0.043571456 | 0.039516188 | CST2/CST4/SPOCK1/CST1 | 4 |
| MF | GO:0004222 | metalloendopeptidase activity | 5/149 | 107/18337 | 0.001811166 | 0.064115291 | 0.058147974 | ADAMTS15/MMP28/MMP7/ADAMTS16/MMP13 | 5 |
| MF | GO:0005539 | glycosaminoglycan binding | 7/149 | 228/18337 | 0.002645292 | 0.085130299 | 0.07720708 | ADAMTS15/SERPINE2/MMP7/F2/CEL/COMP/THBS4 | 7 |
| MF | GO:1901681 | sulfur compound binding | 7/149 | 260/18337 | 0.005420275 | 0.159898115 | 0.145016132 | ADAMTS15/SERPINE2/MMP7/F2/CEL/COMP/THBS4 | 7 |
| MF | GO:0048018 | receptor ligand activity | 10/149 | 486/18337 | 0.006432954 | 0.172368468 | 0.156325849 | FLRT2/SST/F2/NGF/NTS/FGF5/GRP/EPO/IL33/THBS4 | 10 |
| MF | GO:0030546 | signaling receptor activator activity | 10/149 | 492/18337 | 0.006988918 | 0.172368468 | 0.156325849 | FLRT2/SST/F2/NGF/NTS/FGF5/GRP/EPO/IL33/THBS4 | 10 |
| MF | GO:0019841 | retinol binding | 2/149 | 16/18337 | 0.007303749 | 0.172368468 | 0.156325849 | CYP2W1/CYP27C1 | 2 |
| MF | GO:0004867 | serine-type endopeptidase inhibitor activity | 4/149 | 98/18337 | 0.00838336 | 0.18548184 | 0.168218737 | SERPINB7/SERPINA12/SERPINE2/SPOCK1 | 4 |
| MF | GO:0016918 | retinal binding | 2/149 | 18/18337 | 0.009213912 | 0.189514976 | 0.171876502 | CYP2W1/CYP27C1 | 2 |
| MF | GO:0005544 | calcium-dependent phospholipid binding | 3/149 | 54/18337 | 0.009636355 | 0.189514976 | 0.171876502 | ANXA13/CPNE6/DOC2A | 3 |
| MF | GO:0001972 | retinoic acid binding | 2/149 | 20/18337 | 0.011321404 | 0.21093563 | 0.1913035 | CYP2W1/CYP27C1 | 2 |
| MF | GO:0001786 | phosphatidylserine binding | 3/149 | 60/18337 | 0.012834357 | 0.227168115 | 0.206025201 | JPH2/ANXA13/CPNE6 | 3 |
| MF | GO:0048029 | monosaccharide binding | 3/149 | 67/18337 | 0.01724547 | 0.240184543 | 0.217830169 | EGLN3/LMAN1L/HKDC1 | 3 |
| MF | GO:0005104 | fibroblast growth factor receptor binding | 2/149 | 25/18337 | 0.017409226 | 0.240184543 | 0.217830169 | FLRT2/FGF5 | 2 |
| MF | GO:0005227 | calcium activated cation channel activity | 2/149 | 25/18337 | 0.017409226 | 0.240184543 | 0.217830169 | CALHM1/ANO1 | 2 |
| MF | GO:0005179 | hormone activity | 4/149 | 122/18337 | 0.017572679 | 0.240184543 | 0.217830169 | SST/NTS/GRP/EPO | 4 |
| MF | GO:0005518 | collagen binding | 3/149 | 68/18337 | 0.01793626 | 0.240184543 | 0.217830169 | TGFBI/COMP/MMP13 | 3 |
| MF | GO:0005261 | cation channel activity | 7/149 | 333/18337 | 0.019222242 | 0.240184543 | 0.217830169 | CALHM1/CHRNA4/CNGB1/KCNF1/CALHM3/CACNA1B/ANO1 | 7 |
| MF | GO:0033293 | monocarboxylic acid binding | 3/149 | 71/18337 | 0.020100172 | 0.240184543 | 0.217830169 | CYP2W1/CYP27C1/AKR1C2 | 3 |
| MF | GO:0004033 | aldo-keto reductase (NADP) activity | 2/149 | 27/18337 | 0.020154894 | 0.240184543 | 0.217830169 | MIOX/AKR1C2 | 2 |
| MF | GO:0022836 | gated channel activity | 7/149 | 337/18337 | 0.020368632 | 0.240184543 | 0.217830169 | CALHM1/CHRNA4/CNGB1/KCNF1/CALHM3/CACNA1B/ANO1 | 7 |
| MF | GO:0019706 | protein-cysteine S-palmitoyltransferase activity | 2/149 | 28/18337 | 0.021591115 | 0.240184543 | 0.217830169 | ZDHHC11/ZDHHC11B | 2 |
| MF | GO:0019707 | protein-cysteine S-acyltransferase activity | 2/149 | 28/18337 | 0.021591115 | 0.240184543 | 0.217830169 | ZDHHC11/ZDHHC11B | 2 |
| MF | GO:0005244 | voltage-gated ion channel activity | 5/149 | 197/18337 | 0.022482424 | 0.240184543 | 0.217830169 | CALHM1/KCNF1/CALHM3/CACNA1B/ANO1 | 5 |
| MF | GO:0022832 | voltage-gated channel activity | 5/149 | 197/18337 | 0.022482424 | 0.240184543 | 0.217830169 | CALHM1/KCNF1/CALHM3/CACNA1B/ANO1 | 5 |
| MF | GO:0004806 | triglyceride lipase activity | 2/149 | 29/18337 | 0.023068572 | 0.240184543 | 0.217830169 | CES3/CEL | 2 |
| MF | GO:0005184 | neuropeptide hormone activity | 2/149 | 30/18337 | 0.024586508 | 0.248674969 | 0.225530376 | NTS/GRP | 2 |
| MF | GO:0004175 | endopeptidase activity | 8/149 | 438/18337 | 0.026847826 | 0.264003624 | 0.239432368 | PCSK4/ADAMTS15/MMP28/MMP7/F2/ADAMTS16/HPN/MMP13 | 8 |
| MF | GO:0016417 | S-acyltransferase activity | 2/149 | 32/18337 | 0.027740845 | 0.265412411 | 0.240710037 | ZDHHC11/ZDHHC11B | 2 |
| MF | GO:0005506 | iron ion binding | 4/149 | 150/18337 | 0.03413227 | 0.317969046 | 0.288375139 | MIOX/CYP2W1/CYP27C1/EGLN3 | 4 |
| MF | GO:0005501 | retinoid binding | 2/149 | 37/18337 | 0.036283992 | 0.329347 | 0.298694128 | CYP2W1/CYP27C1 | 2 |
| MF | GO:0019840 | isoprenoid binding | 2/149 | 38/18337 | 0.038099679 | 0.337182163 | 0.305800058 | CYP2W1/CYP27C1 | 2 |
| MF | GO:0016409 | palmitoyltransferase activity | 2/149 | 39/18337 | 0.03994946 | 0.338487864 | 0.306984236 | ZDHHC11/ZDHHC11B | 2 |
| MF | GO:0072341 | modified amino acid binding | 3/149 | 93/18337 | 0.040159577 | 0.338487864 | 0.306984236 | JPH2/ANXA13/CPNE6 | 3 |
| MF | GO:0016705 | oxidoreductase activity, acting on paired donors, with incorporation or reduction of molecular oxygen | 4/149 | 160/18337 | 0.041674251 | 0.340525263 | 0.30883201 | CYP2W1/CYP27C1/EGLN3/AKR1C2 | 4 |
| MF | GO:0008238 | exopeptidase activity | 3/149 | 95/18337 | 0.042343808 | 0.340525263 | 0.30883201 | TRHDE/CPA2/HPN | 3 |
| MF | GO:0008083 | growth factor activity | 4/149 | 162/18337 | 0.04328711 | 0.340525263 | 0.30883201 | F2/NGF/FGF5/THBS4 | 4 |
| MF | GO:0022839 | ion gated channel activity | 2/149 | 42/18337 | 0.045696741 | 0.351666227 | 0.318936066 | CALHM1/ANO1 | 2 |
| MF | GO:0004252 | serine-type endopeptidase activity | 4/149 | 168/18337 | 0.048334543 | 0.354612489 | 0.321608115 | PCSK4/MMP7/F2/HPN | 4 |
| MF | GO:0004497 | monooxygenase activity | 3/149 | 101/18337 | 0.049246304 | 0.354612489 | 0.321608115 | CYP2W1/CYP27C1/AKR1C2 | 3 |
| MF | GO:0005245 | voltage-gated calcium channel activity | 2/149 | 44/18337 | 0.049686743 | 0.354612489 | 0.321608115 | CALHM1/CACNA1B | 2 |
