# Supplementary material for: The prognostic value of tumor mutational burden related 6-gene-based Risk Score in laryngeal cancer patients
Source: BMC Oral Health. 2022 Nov 17;22:510. doi: 10.1186/s12903-022-02534-2 (PMC9673449; doi:10.1186/s12903-022-02534-2)
Supplement: Supplementary file 2 — Additional file 2: Table S2. Full results of KEGG enrichment analysis of 210 DEGs. [file 12903_2022_2534_MOESM2_ESM.docx]

**Table S2 Full results of KEGG enrichment analysis of 210 DEGs**

| ID | Description | GeneRatio | BgRatio | pvalue | p.adjust | qvalue | geneID | Count |
| --- | --- | --- | --- | --- | --- | --- | --- | --- |
| hsa04610 | Complement and coagulation cascades | 4/63 | 85/8096 | 0.004210207 | 0.25507658 | 0.246126524 | 10877/7056/2147/730 | 4 |
| hsa04080 | Neuroactive ligand-receptor interaction | 8/63 | 342/8096 | 0.004752781 | 0.25507658 | 0.246126524 | 1137/147/6750/2147/4922/5618/2922/23566 | 8 |
| hsa04970 | Salivary secretion | 4/63 | 93/8096 | 0.005797195 | 0.25507658 | 0.246126524 | 1470/147/1472/1469 | 4 |
| hsa04151 | PI3K-Akt signaling pathway | 7/63 | 354/8096 | 0.019427354 | 0.531757456 | 0.5130993 | 4803/1311/5618/2250/2056/23566/7060 | 7 |
| hsa00052 | Galactose metabolism | 2/63 | 31/8096 | 0.023974329 | 0.531757456 | 0.5130993 | 3938/80201 | 2 |
| hsa05207 | Chemical carcinogenesis - receptor activation | 5/63 | 212/8096 | 0.024170793 | 0.531757456 | 0.5130993 | 1137/2940/79852/774/2250 | 5 |
| hsa04512 | ECM-receptor interaction | 3/63 | 88/8096 | 0.030864223 | 0.535106819 | 0.516331141 | 9899/1311/7060 | 3 |
| hsa04020 | Calcium signaling pathway | 5/63 | 240/8096 | 0.038357143 | 0.535106819 | 0.516331141 | 6543/147/4803/774/2250 | 5 |
| hsa05033 | Nicotine addiction | 2/63 | 40/8096 | 0.038464545 | 0.535106819 | 0.516331141 | 1137/774 | 2 |
| hsa04974 | Protein digestion and absorption | 3/63 | 103/8096 | 0.045857064 | 0.535106819 | 0.516331141 | 169044/6543/1358 | 3 |
| hsa04930 | Type II diabetes mellitus | 2/63 | 46/8096 | 0.049553876 | 0.535106819 | 0.516331141 | 774/80201 | 2 |
